# Temporal Non-Local Means Filtering Reveals Real-Time Whole-Brain Cortical Interactions in Resting fMRI

**DOI:** 10.1371/journal.pone.0158504

**Published:** 2016-07-08

**Authors:** Chitresh Bhushan, Minqi Chong, Soyoung Choi, Anand A. Joshi, Justin P. Haldar, Hanna Damasio, Richard M. Leahy

**Affiliations:** 1 Signal and Image Processing Institute, University of Southern California, Los Angeles, CA, United States of America; 2 Neuroscience Graduate Program, University of Southern California, Los Angeles, CA, United States of America; 3 Brain and Creativity Institute, Dornsife College of Letters, Arts and Sciences, University of Southern California, Los Angeles, CA, United States of America; National Scientific and Technical Research Council (CONICET)., ARGENTINA

## Abstract

Intensity variations over time in resting BOLD fMRI exhibit spatial correlation patterns consistent with a set of large scale cortical networks. However, visualizations of this data on the brain surface, even after extensive preprocessing, are dominated by local intensity fluctuations that obscure larger scale behavior. Our novel adaptation of non-local means (NLM) filtering, which we refer to as temporal NLM or tNLM, reduces these local fluctuations without the spatial blurring that occurs when using standard linear filtering methods. We show examples of tNLM filtering that allow direct visualization of spatio-temporal behavior on the cortical surface. These results reveal patterns of activity consistent with known networks as well as more complex dynamic changes within and between these networks. This ability to directly visualize brain activity may facilitate new insights into spontaneous brain dynamics. Further, temporal NLM can also be used as a preprocessor for resting fMRI for exploration of dynamic brain networks. We demonstrate its utility through application to graph-based functional cortical parcellation. Simulations with known ground truth functional regions demonstrate that tNLM filtering prior to parcellation avoids the formation of false parcels that can arise when using linear filtering. Application to resting fMRI data from the Human Connectome Project shows significant improvement, in comparison to linear filtering, in quantitative agreement with functional regions identified independently using task-based experiments as well as in test-retest reliability.

## Introduction

Low frequency fluctuations in BOLD activity during resting functional MRI (rfMRI) exhibit correlations between cortical regions that are known to be physiologically related, as first shown by Biswal et al. [1, 2]. These correlations are the basis for identification of functional networks from rfMRI in individuals and groups [2–5]. These rfMRI data are typically preprocessed prior to network analysis with a pipeline that includes compensation for susceptibility-induced distortion, slice timing and subject motion, as well as high-pass filtering of individual time series and removal of ICA-identified temporal noise components [6–8]. Even after this extensive preprocessing, when visualized as a time series or movie of cortical activity, correlated patterns of BOLD variation reflecting time-varying brain activity are not readily visible in the data. Local variations in network-related BOLD activity and unrelated physiological and other noise in the data mask these underlying patterns.

To reduce noise in fMRI it is common to spatially smooth the data, typically with an isotropic kernel applied in the volumetric space [4–6, 9]. Isotropic 3D linear smoothing will inevitably mix signals from areas that are not directly adjacent with respect to cortical geometry, for example blurring across the void from one side of a sulcal bank to the other. To avoid this problem, data can be smoothed directly in the 2D manifold of the cortical surface. This is achieved using the Laplace-Beltrami operator that accounts for local surface curvature to generalize the 2D Gaussian smoothing kernel to an arbitrary smooth manifold [10].

While Laplace-Beltrami (LB) smoothing can avoid blurring across sulcal banks, the smoothing is still linear and isotropic so that sharp spatial features in the functional data are smoothed, blurring boundaries on the cortical surface between distinct functional regions. As we demonstrate in our simulations below, the resulting signal mixing can also confound cortical parcellation methods, introducing artifactual parcels purely as a result of isotropic smoothing. The primary contribution of this paper is to describe an alternative nonlinear filtering method based on a novel adaptation of non-local means that reduces noise while also respecting functional boundaries. The results presented below all use rfMRI data resampled onto the cortical surface, but the filtering method we describe can also be applied directly to volumetric data.

Non-local means (NLM) is an edge-preserving filtering method that uses the weighted average of pixels in a large neighborhood where these weights are chosen adaptively depending on the structural similarities in the local neighborhoods of each pixel [11]. As a result NLM reduces noise while simultaneously retaining spatial structure by averaging only over pixels that have similar local structure. NLM filtering has previously been applied to structural [12–14], functional [15–17], and diffusion [18, 19] MRI data. Modified NLM methods tailored to MRI data have also been developed including block-wise filtering and automatic adaption of weights based on SNR [12, 13, 19], multi-component extensions [14], and use of multiple angular components for HARDI MRI [18]. All of these approaches use spatial similarity over one or more images as the basis for NLM smoothing. While this approach can be applied to fMRI time series [15–17], filtering each temporal frame separately with NLM will produce a time-varying smoothing kernel, confounding subsequent time-series analysis.

We have developed a novel variation on NLM which we refer to as temporal NLM (tNLM). Our method directly exploits the temporal information in the data by replacing the standard spatial similarity weighting in NLM with a weighting that is based on the correlation between time series. As a result we reduce noise by averaging only those pixels that have similar time series. This prevents smoothing across functional boundaries, since the time series within distinct functional regions will be more strongly correlated than those in different functional areas. A related approach was used by one of the authors for denoising dynamic PET data by combining local spatial and temporal information to compute NLM weights [20].

In addition to demonstrating the impact of tNLM in terms of revealing spatio-temporal structure in rfMRI data, we also illustrate its utility through one application: functional cortical parcellation. A number of parcellation methods have recently been described that include spectral clustering, hierarchical clustering, edge detection, and snow-balling [3–5, 9, 21]. Our goal here is to investigate the effect of tNLM filtering on cortical parcellation of rfMRI data and compare performance with unfiltered and isotropically filtered data. For this purpose we use a spectral clustering method based on normalized cuts [22, 23], although other parcellation methods could also be used [3, 21]. Our evaluations with simulations and experimental *in vivo* human data demonstrates meaningful subdivisions, significantly improved consistency in test-retest evaluations and better agreement with regions identified with independent task-based experiments.

## Materials and Methods

### Dataset and preprocessing

All of the results in this report and the supporting information used the minimally preprocessed (ICA-FIX denoised) rfMRI data from 40 unrelated subjects, which are publicly available from the Human Connectome Project (HCP) [24]. The data were resampled onto the individuals’ cortical surfaces. All analysis we describe was performed with respect to these surface representations. Here we briefly describe the HCP dataset and preprocessing; more details can be found in references [6, 7, 24–26] and in section A in S1 Text. The functional MRI data sets were acquired for four independent resting state sessions of 15 minutes each (TR = 720ms, TE = 33.1ms, 2 × 2 × 2 mm voxel). The subjects were asked to relax and fixate on a projected bright cross-hair on a dark background [24]. HCP’s minimal preprocessing primarily corrects the rfMRI data for acquisition artifacts, resamples the data onto the cortical surface and performs a non-aggressive spatio-temporal cleanup [6, 7]. The artifact correction step allows compensation for head motion and spatial distortion caused by gradient non-linearity and B_0_ field inhomogeneity. The corrected functional data is then co-registered with the corresponding structural images and resampled onto the 32K Conte-69 cortical mesh in the native subject space [6, 27]. Next, spatio-temporal processing is used to remove the residual effect of scanner and motion artifacts and non-neuronal physiological artifacts, this includes a weak high-pass temporal filtering (no low-pass filtering) followed by regressing out the artifactual temporal time-courses identified using ICA-FIX on the volumetric data [6]. The only additional preprocessing step we introduced prior to tNLM or LB filtering was to normalize the time series associated with each cortical vertex to zero mean and unit variance.

The functional parcellation results presented below are also evaluated using the task-localizer data made available by the HCP. Task-based fMRI data were obtained for the same 40 subjects and included six major task domains: somatosensory and motor systems, language processing, social cognition, relational processing, emotion processing and decision making [25, 26]. We used HCP’s pre-processed and analyzed task-fMRI data resampled onto the cortical surface, with different levels of Gaussian smoothing (described below), which yielded a total of 17 statistical task-pair activation maps [25, 26]: (1) Faces vs. Shapes, (2) Shapes vs. Faces, (3) Punish vs. Reward, (4) Reward vs. punish, (5) Math vs. Story, (6) Story vs. Math, (7) Left foot, (8) Left hand, (9) Right foot, (10) Right hand, (11) Tongue, (12) Match vs. Rel., (13) Rel. vs. Match, (14) Random vs. Tom, (15) Tom vs. random, (16) 0-back vs. 2-back, and (17) 2-back vs. 0-back. Additional comparisons of our rfMRI parcellation results with probabilistic Brodmann areas are included in S1 Text.

### Temporal non-local means (tNLM)

Non-local means (NLM) is a widely used technique for edge-preserving filtering of images [11]. In common with conventional linear filtering, NLM uses weighted spatial averaging to reduce noise. However rather than using a set of spatially invariant weights applied to pixels in a local neighborhood, the NLM weights are based on a measure of similarity of a small neighborhood, or a patch, surrounding each pixel [11]. When the patches around two pixels are similar the weight is large; and when they are dissimilar, the weight is low. In this way, the weighted averaging tends to reinforce spatial structure while removing noise.

In this work, we are interested in identifying functional regions that share common temporal variations. For this reason tNLM uses a weight based on the similarity of the time series, rather than a spatial patch, to filter the data. Specifically, let *d*(*s*, *τ*) denote the rfMRI data at surface vertex *s* at time *τ*. Then the corresponding tNLM-filtered rfMRI is given by

$$\begin{array}{c} f\left(s,\tau \right)=\frac{1}{\sum_{r\in N(s)}w\left(s,r\right)}\sum_{r\in N(s)}d\left(r,\tau \right)\;w\left(s,r\right)\;, \end{array}$$ (1)

where N(s) denotes a set of vertices on the tessellated cortical surface lying in a large neighborhood surrounding vertex *s* and *w*(*s*, *r*) is the weight applied to vertex r∈N(s) when filtering the rfMRI data at vertex *s*. We parameterize the neighborhood of a vertex on the cortical mesh by the linked distance parameter *D* such that the set N(s) contains all vertices, including itself, which are at a linked distance of *D* or less from the vertex *s* (we use *D* = 11 in all our results as it shows empirically good results with reasonable computational cost). The weights *w*(*s*, *r*) are given by

$$\begin{array}{c} w\left(s,r\right)=\exp(-\frac{\frac{1}{T}{\left|\right|\underline{\text{d}}\left(s\right)-\underline{\text{d}}\left(r\right)\left|\right|}^{2}}{h^{2}}) \end{array}$$ (2)

where $\underline{\text{d}}\left(s\right)={\left[d(s,1),\cdots ,d(s,T)\right]}^{⊤}$ is a vector of length *T* representing the time series at vertex *s* and *h* is scalar parameter which determines the rate at which the weights decrease with decreasing similarity between the two time series. Because we pre-process the time series at each vertex to have zero mean and unit variance, the weights in Eq (2) are equivalent to using Pearson’s correlation coefficient $\text{corr}(\underline{\text{d}}\left(s\right),\underline{\text{d}}\left(r\right))$ between $\underline{\text{d}}\left(s\right)$ and $\underline{\text{d}}\left(r\right)$, since $\frac{1}{T}{\left|\right|\underline{\text{d}}\left(s\right)-\underline{\text{d}}\left(r\right)\left|\right|}^{2}=2-2\times \text{corr}(\underline{\text{d}}\left(s\right),\underline{\text{d}}\left(r\right))$.

In the following, we compare tNLM with linear filtering on the cortical surface. As noted in the introduction, linear filtering directly on the cortical surface is preferable to volumetric smoothing (prior to resampling on the cortical surface) as this avoids blurring of data across sulcal banks. We use the Laplace-Beltrami (LB) operator for filtering, which is a generalization of 2D Gaussian filtering that accounts for surface curvature to perform isotropic smoothing of data defined on that surface [10]. A single parameter *t* controls the degree of smoothing and we use a truncated eigenfunction expansion to efficiently perform LB filtering [28–30] (see S1 Text for details).

### Identification of cortical networks

To explore the impact of LB and tNLM filtering on cortical parcellation we used a graph-based spectral clustering method to identify a set of functional networks for each subject. We represent the spatio-temporal rfMRI data as a graph *G* = (*V*, *A*) where the set of vertices of the cortical tessellation are the nodes *v* ∈ *V* of the graph and *A* is the adjacency (edge) matrix such that any two vertices *u*, *v* ∈ *V* are connected by an undirected edge of strength $A\left(u,v\right)=\exp({\underline{\text{d}}}^{⊤}\left(u\right)\underline{\text{d}}\left(v\right)/T)$.

The normalized-cuts (N-cuts) algorithm [22] subdivides the graph *G* into *K* sub-graphs by subdividing the nodes (or vertices on the tessellated cortical surface), *V*, into *K* disjoint subsets *V*_1_, *V*_2_, ⋯, *V*_*K*_ so that $\cup_{i=1}^{K}V_{i}=V$ and $\forall i\neq j,\;V_{i}\cap V_{j}=⌀$. N-cuts partitions the graph to maximize the average “normalized association” within each of the *K* sub-graphs, which can be expressed as the cost function:

$$\begin{array}{c} \text{Nassoc}(\{V_{1},\cdots ,V_{K}\})=\frac{1}{K}\sum_{i=1}^{K}(\frac{\sum_{u,v\in V_{i}}A\left(u,v\right)}{\sum_{u\in V_{i},v\in V}A\left(u,v\right)}) \end{array}$$ (3)

Yu and Shi [22, 23] show this cost is equivalent to minimizing the average normalized cut cost. N-cuts therefore finds the set of *K* sub-graphs that have the weakest normalized average connectivity between sub-graphs and the maximum connectivity within each sub-graph. In this paper, we use the implementation of N-cuts provided by the authors (from http://www.cis.upenn.edu/~jshi/software).

Note that the graph definition described above produces a fully connected graph that contains no explicit spatial information about each vertex’s neighborhood structure. It is common to explicitly introduce spatial neighborhood information for functional clustering of rfMRI data, typically by restricting the graph-edges to pairs of spatially neighboring vertices, making the adjacency matrix *A* sparse [3–5]. In preliminary evaluations (not shown) we found that the fully connected graph produced more reliable parcellation with tNLM, presumably because the fully connected graph contains far more information about functional similarity of nodes/vertices than the sparser spatially constrained graph.

The final result of the N-cuts partitioning of the graph is a disjoint set of subgraphs, each containing a subset of cortical vertices that can be interpreted as jointly participating in a single functional network or subnetwork. Since the subgraphs are not constrained to be spatially adjacent, each subgraph can contain multiple disjoint cortical regions or patches that make up the network. The boundaries of these regions form the cortical parcellations we show below.

### Performance evaluation

We compare the performance of tNLM and LB filtering using a variety of qualitative and quantitative methods. In this section, we describe the technical details for different approaches for evaluating the effect of filtering on rfMRI. The corresponding results are presented in the next section.

#### N-cuts networks: parameters, visualization and boundaries

We studied the effect of filtering rfMRI data on cortical parcellation by comparing the classification of cortical networks using N-cuts with a large range of parameters. The N-cuts clustering approach sub-divides all the vertices into *K* disjoint sets as described previously. We assign a unique label ID to all vertices which are clustered in the same set and visualize them with a unique color on the cortical surface (so that a clustering result with *K* classes will have *K* unique colors). We performed N-cuts classification, with several values of *K*, on a total of 160 *in vivo* rfMRI dataset (40 subjects × 4 sessions) without filtering and with LB and tNLM filtering. For both filtering types, we present results with three different level of smoothing: *h* = 0.60, 0.72, 1.73 for tNLM and *t* = 2, 4, 10 for LB filtering. The range of parameter *h* for tNLM was chosen based on visual inspection of the resulting smoothed data and a preliminary performance study. For qualitative comparisons of tNLM and LB filtering, we choose values of the LB parameter *t* by approximately matching the mutual information of LB filtered results with tNLM results for each value of *h*. Based on the preliminary study we chose a value of *h* = 0.72 for tNLM filtering for the majority of the qualitative results presented below (the corresponding LB parameter was found to be *t* = 4). We note that the nature of smoothing with tNLM and LB is very different and hence matching of smoothing levels/parameters across tNLM and LB only serves qualitative purposes. Our quantitative evaluations, described later, compare parcellation performance across all parameter values.

In order to enable color-coded visualization and comparisons of cortical parcellations obtained with a fixed value of *K* but with different filtering approaches or subjects, we first identify equivalent sub-networks by using the parcellation matching method described in Sec. C in S1 Text, which is based on the Gale-Shapley stable matching algorithm [31]. The matching method establishes a one-to-one matching between two parcellations allowing us to use the same color to represent equivalent parcels across subjects or methods.

We also investigated how the boundaries of functional regions changed with the number of networks *K* and the filtering approach used. For each parcellation, we obtained a binary boundary map by defining a triangle on the tessellated cortical mesh as a ‘boundary’ triangle if its vertices lie in more than one subgraph. We summarized the boundaries across values of *K* = 2, 3, 4, 5, 6, 7, 8, 9, 10, 15, 30, 40, 50, 60, 80 by computing the cumulative boundary map across parcellations obtained with these values of *K* for each filtering approach and each subject. In the cumulative boundary map, the value at each triangle represents the number of times that triangle has been identified as a boundary triangle across all values of *K*. We also summarized the cumulative boundary maps across population by computing the population average across 40 subjects for both tNLM and LB filtering.

#### Agreement with task activation labels

As no ground-truth parcellation is available for *in vivo* data, we evaluated the quality of the functional parcellations through quantitative comparisons with task activations for each subject. Task-based experiments allow delineation of different functional areas in cortex and have previously been shown to have close correspondence to those identified using rfMRI [1, 2]. Hence, a “good” resting fMRI parcellation should obtain high agreement with task-labels identified using independent task-based fMRI. We used statistical activation maps for each of the 17 different task-pairs described earlier, which are available for all 40-subjects from HCP, to obtain a discrete task label map for each subject.

We first thresholded each task-pair activation map at Z-score ≥ 3.0 (one-tailed uncorrected p-value ≤ 0.00135) and merged them into a single cortical map with a unique label for each task-pair. If a vertex has Z-score ≥ 3.0 in more than one task-pair activation map then we assigned the task label corresponding to the most significant activation. The labeled map was then cleaned by removing isolated labeled patches of size 40 vertices or less to obtain the final task label map for each subject, which was used as the comparison reference for rfMRI parcels. Only nine task-pairs survived statistical and spatial thresholding and hence we only present results with these task-pairs. Also note that the task-fMRI data was pre-analyzed with different levels of Gaussian smoothing (FWHM of 2mm, 4mm, and 8mm) to produce three different task-activation maps. Here, we present results with FWHM of 4mm, however results with all other levels of smoothing are included in S1 Text.

We computed label-wise agreement between the task label maps and N-cuts parcellation using the matching method described in section C in S1 Text (we use task label as *A* and N-cuts parcellation as *B*). The agreement measure is defined as the fraction of vertices for each task label that correspond to the N-cuts parcel to which that task is matched. We computed the label-wise agreement measure separately for each parcellation, and used these measures to compare across filtering methods and parameters.

## Results

### Visualization of brain activity from rfMRI signal

**Fig 1:**
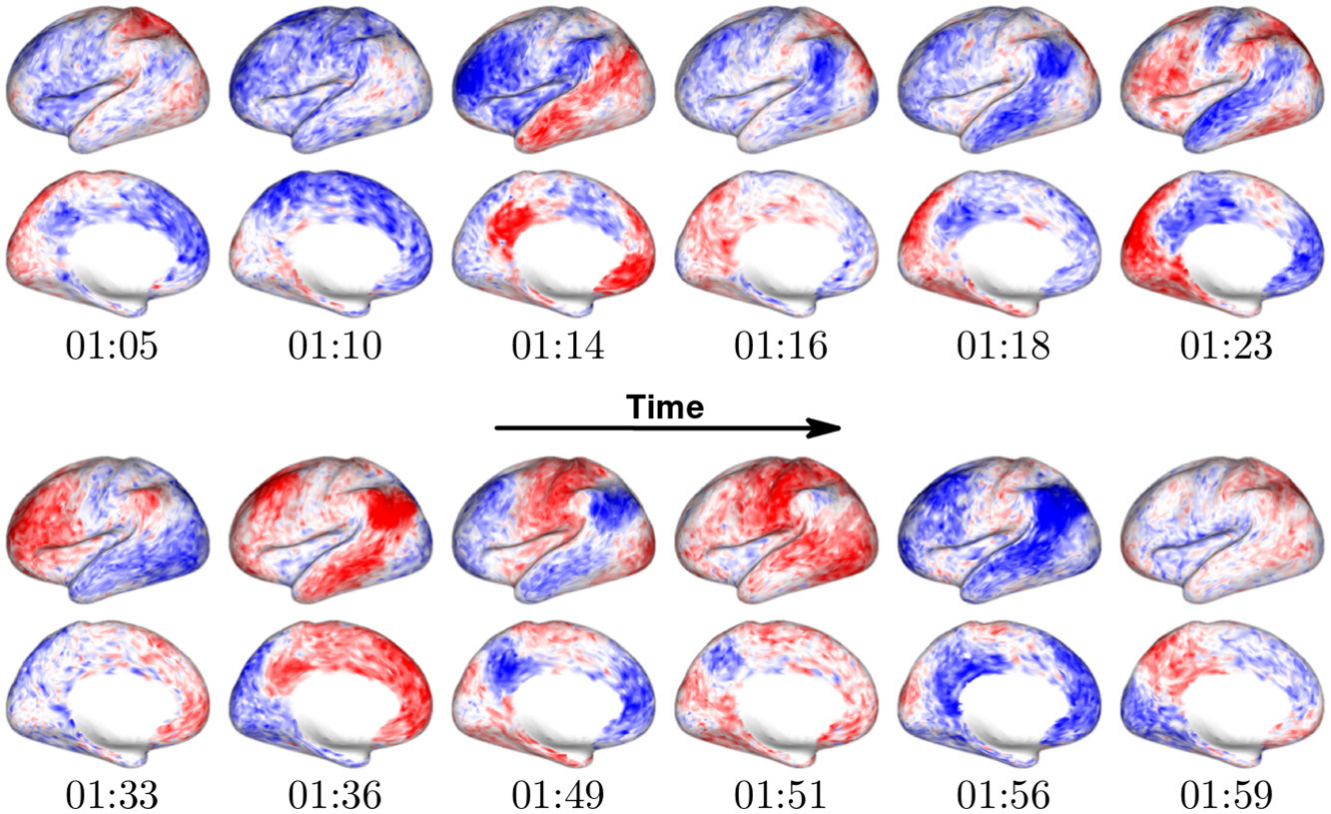
Representative frames, taken from S1 Video, illustrating dynamic brain activity at “rest”, as seen with tNLM filtering (*h* = 0.72). See S1 Video for the complete 2.5 min movie. Each subfigure shows the BOLD intensity in left-hemisphere at a particular time-point after tNLM filtering of a 15-minutes long minimally processed rfMRI data. The brain activation regions shift dynamically from one network to another, which can be most easily noticed around the default mode network (DMN) and anti-correlated DMN. These networks consist predominantly of large regions distributed throughout the brain that are spatially separate but have near synchronous temporal activity. At 01:05, we see activity below the mean in the DMN with the rest of the brain showing mostly activity above the mean. By 1:14 we see the opposite brain activity pattern where the DMN is now above the mean. The rest of the brain shows mostly activity below the mean, with the exception of the upper half of the sensory-motor cortices (SMC) which, on the right, show some activity above the mean, mostly mesially. Only 2 seconds later, at 01:16, the lateral temporal and parietal nodes of the DMN show activity clearly below the mean, while the activity in the PMC is still above the mean, but less so, and the activity in the mesial frontal regions is now mostly below the mean; another 2 seconds later, at 01:18, all of the DMN nodes are clearly below the mean, while mesial occipital regions are above the mean; five more second have passed (01:23) and the image is almost the reverse of what was seen at 01:14; the DMN nodes show clear negativity, as does SMC, while the rest of the brain, including the insulae, is above the mean; after 10 seconds, at 01:33, the frontal lobe, a small area of the SMC and the insula are above the mean, while the remainder of the hemisphere is below the mean; after another 3 seconds, at 01:36, the DMN nodes are well above the mean (more so than at 01:45), the occipital lobe below the mean, and the insula and dorsolateral SMC close to the mean, but the mesial motor cortices well above the mean; at 01:49 the DMN nodes are negative while the SMC shows activity clearly above the mean, and so do the occipital lobe and the insula; two seconds later, at 01:51, the brain activity is in general above the mean with three interesting exceptions: the PMC, the angular gyrus and the insula show activity below the mean; five seconds later, at 01:56, the brain is massively negative with a few exceptions where the activity approximates the mean; and at 01:59 the DMN nodes again start to show activity above the mean.

**Fig 2:**
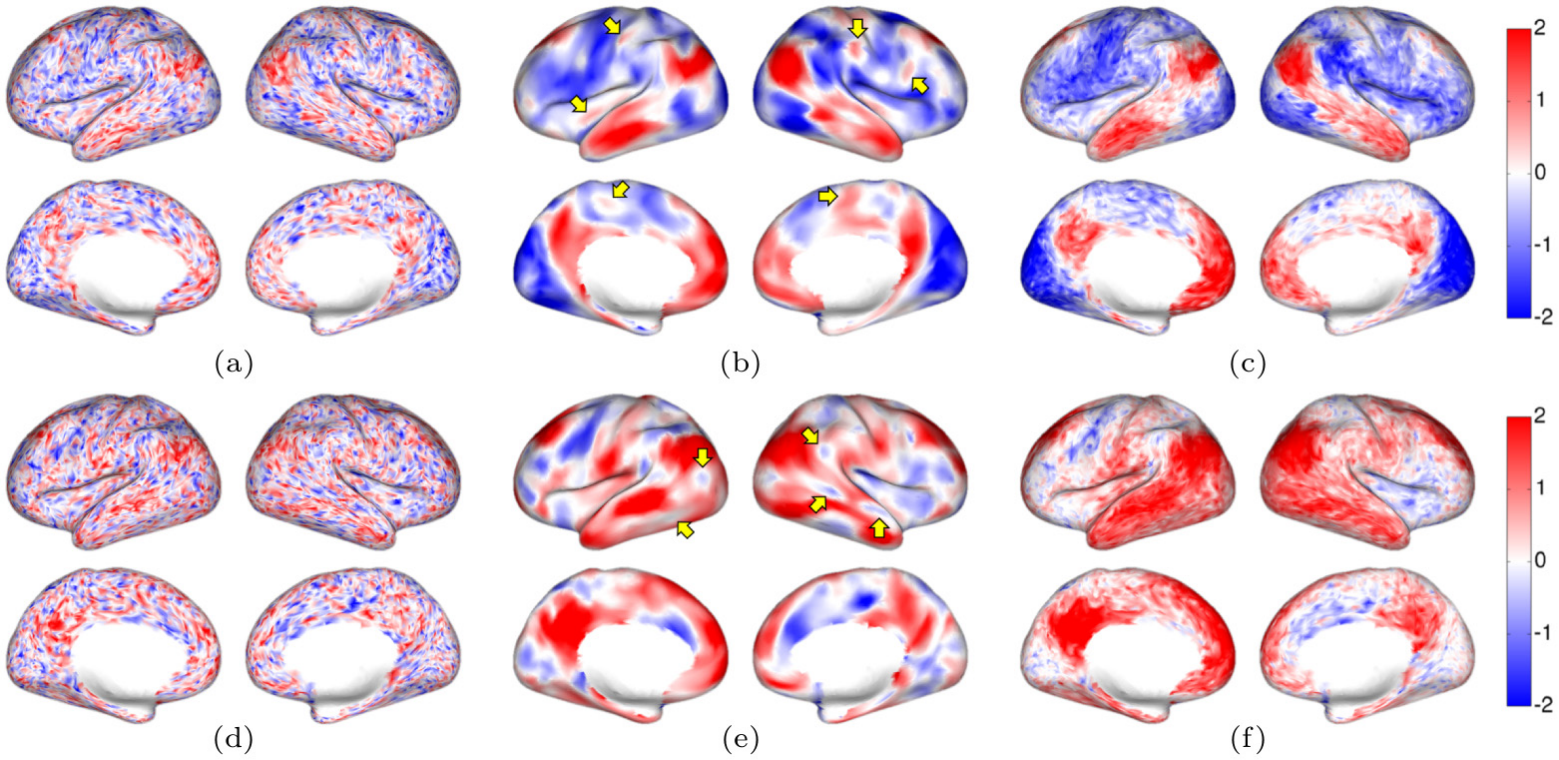
Illustration of smoothing effects on cortical BOLD signal intensity in rfMRI in a single subject, shown at a single time point: (a) no filtering, (b) LB filtering (*t* = 4) and (c) tNLM filtering (*h* = 0.72). Color scale shows positive (red), negative (blue) and zero (white) BOLD signal intensity. 2.5 minute real-time movies showing the un-filtered, LB- and tNLM-filtered rfMRI data can be found in the Supplemental Information (S1–S3 Videos). It is difficult to detect spatial structure in the original unfiltered data, even if there are hints that can be discerned. By applying either LB or tNLM filtering, however, the noise is reduced and coherence in local activation/deactivation with respect to the underlying anatomy of the cerebral cortex is revealed. We see synchronous bilateral activity (in red) for both filtering methods in brain regions associated with the anterio-medial, posterio-medial and dorso-lateral regions of the DMN. LB filtering (b) however, shows some additional small isolated patches in the fronto-lateral cortex, anterior insula, and the post-central gyri and the mesial motor regions, as indicated by the arrows. Interestingly, most of these isolated patches lie in regions that have been reported to show strong negative correlations to the DMN [32–35], and so are unlikely to be synchronous with DMN regions. Similar behavior can be observed at another time point when (d) the original rfMRI data is filtered with (e) LB and (f) tNLM, where most of the DMN regions again show synchronous BOLD signal intensity in red. The tNLM results appear clearer in the sense that contrast in the images and movies appears to more closely follow discrete anatomical regions than do the LB results. The arrows in (e) show small regions of activation/deactivation in the LB filtered data that may result from smoothing across distinct functional areas. These may subsequently give rise to erroneous parcellation results as described in the text. The differences between the two methods is more readily evident in the movies of continuous resting state recording (see S1–S3 Videos). Note in particular the different dynamic of the changes in brain activity—LB filtered images change smoothly from one brain state to the next while the tNLM images depict a more burst like change across consecutive brain states.

The qualitative impact of tNLM filtering on rfMRI data is best seen in the accompanying movie (S1 Video) from which we also show a series of still frames in Fig 1. For reference we also include videos of the unfiltered (S2 Video), and LB filtered (S3 Video) data, as well as a comparison of sample frames in Fig 2.

The movies represent the BOLD signal intensities mapped onto a smoothed representation of the cortical surface and were made by concatenating individual images spaced by TR = 720ms and linearly interpolated to 10 frames per second. The movies are generated at a real-time frame rate, *i.e.* they show 2.5 minutes of BOLD activity played back over 2.5 minutes, and were encoded using the mp4 H.264 codec. The signal intensities in the movies and Fig 1 use the same color map as Fig 2, with transitions from blue (negative) to white (zero) to red (positive). As noted above, the BOLD time series at each surface vertex were normalized to zero mean, unit variance before filtering, resulting in the dynamic range shown of approximately ±2.

In Fig 1 we show examples of images obtained at different time points with tNLM filtering with detailed descriptions in the legends. Images at corresponding time points are shown in Fig A in S1 Text for unfiltered data and LB smoothing. In Fig 2 we show the cortical BOLD signal for a single subject before and after smoothing using LB and tNLM. The effect of different degrees of smoothing (varying parameters *h* in tNLM and *t* in LB) is shown in Fig B in S1 Text. As described in the legends, Figs 1 and 2 illustrate the qualitative differences between unfiltered and tNLM and LB filtered data. LB filtering does not use information about the time course to filter at each point in time and so can mix signals across neighboring vertices with very dissimilar time courses; tNLM filtering on the other hand uses weights based on similarity of the time series and so can avoid mixing of signals from dissimilar vertices. These differences are reflected qualitatively in both the movies and figures, with the tNLM images showing more apparent consistency over time in the location of boundaries between regions exhibiting differing dynamic behavior. In contrast LB movies and images show smaller scale time-varying features (indicated by arrows in Fig 2)that are consistent with mixing of functionally distinct regions. We explore this issue further in the following simulation.

### Simulation: Effect of smoothing on clustering

**Fig 3:**
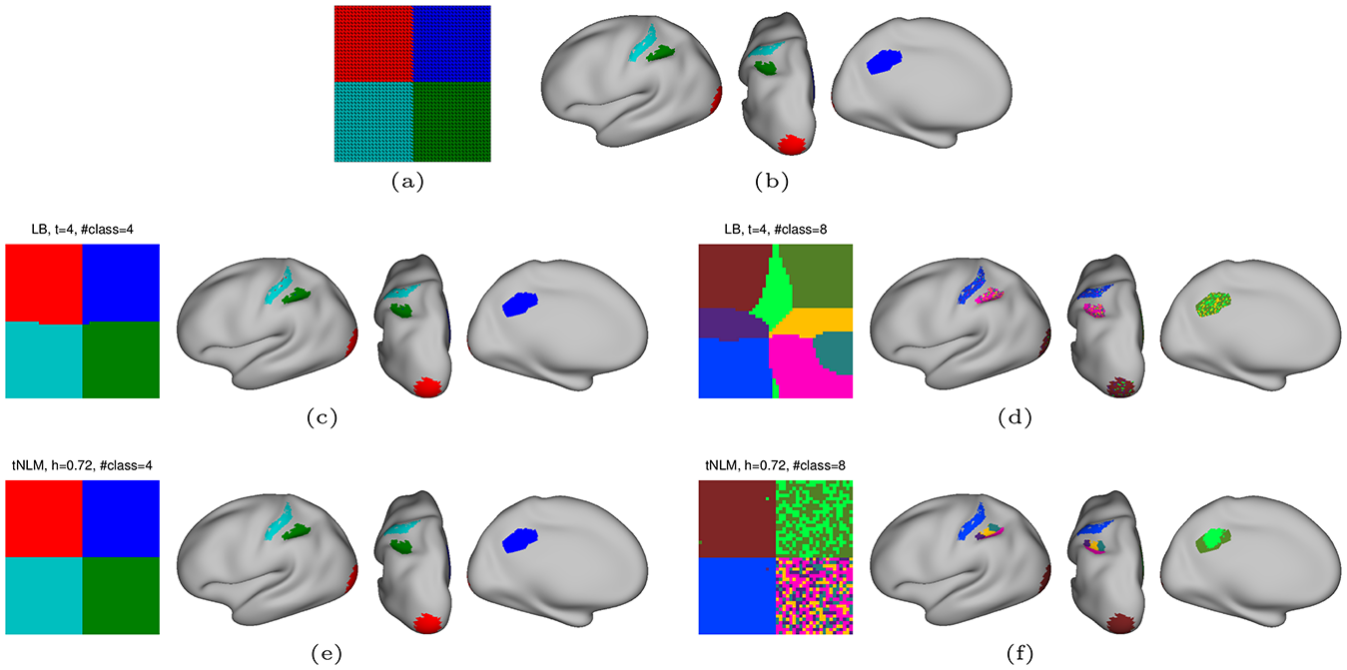
Effect of smoothing on simulated surface with known functional regions. (a) The square surface on which smoothing and parcellation is performed. The four quadrants are color-coded for easy identification and represents functionally distinct regions. (b) The location of the four regions on cortex from which the time series for each quadrant are drawn. The result of N-cuts parcellation of LB filtered data into (c) *K* = 4 and (d) *K* = 8 clusters are also shown on the simulated square surface as well as on the original cortex, on the right, by mapping back the color-coded vertices from the square mesh to its original location on the cortex. Similarly, the results of N-cut parcellation for tNLM filtered data into (e) *K* = 4 and (f) *K* = 8 clusters are shown on square surface and on the original surfaces.

We used a simulated data set to investigate the effect of tNLM and LB filtering on N-cuts based identification of functionally distinct cortical regions. We generated a square surface patch with four quadrants, where each quadrant was simulated to correspond to a different functionally distinct region. Vertices in each quadrant were assigned a set of rfMRI time series data drawn from an equal number of vertices in small cortical regions in one of the HCP rfMRI data sets from a single subject. These regions were chosen to lie in well-known prominent networks (visual, motor, default mode and task positive) such that each region was functionally distinct from other regions and was locally homogeneous, based on the mean correlation with neighboring vertices. The correspondence between the four quadrants of the simulated surface and the locations on the cortex from which each quadrant was sampled are shown in Fig 3(a) and 3(b). This simulated surface, with known location of functional regions, provides an avenue to study the effect of filtering on the accuracy of parcellation.

We identified networks in the four quadrant rfMRI data using N-cuts based parcellation, as described earlier, to find *K* = 4 and *K* = 8 networks in the original data and after filtering with LB and tNLM. Note that the N-cuts parcellation method described above uses a fully connected graph with edge strength based on time-series similarity and therefore spatial proximity between vertices is not directly encoded in the graph structure. The parcellation results are almost perfect for *K* = 4 in both cases (Fig 3(c) and 3(e)) and, indeed, also were for the unfiltered data (not shown) since the four regions were chosen to be internally homogeneous with respect to their time series and with a low or negative correlation between the time series in different regions. However, the clustering with *K* = 8 produces very different results as shown in Fig 3(d) and 3(f). The linear mixing across quadrant boundaries using LB filtering produces intermediate regions that internally have a higher correlation than they do with the two regions from which the data originated. The resulting parcellation therefore includes new contiguous regions at the boundaries between quadrants that were not present in the original data (Fig 3(d), clusters in light-green, yellow and violet). When these clusters are mapped back to the surface vertices on the cortex from which they were drawn, we see they appear distributed across more than one functional area (for example elements in the light-green parcel appear in DMN, visual and motor areas). This clearly demonstrates that the contiguous regions at the boundaries between quadrants found in the LB filtered data are solely an artifact of mixing from LB smoothing and do not reflect true underlying patterns of functional similarity in the rfMRI time series. We believe this in an important observation as the creation of false parcels resulting from LB smoothing could lead to erroneous interpretation of parcellations in *in vivo* data.

In contrast, tNLM filtering shows a strong pattern of sub-division of functionally distinct areas when *K* = 8 class clustering was used, Fig 3(f): vertices in the top right quadrant (corresponding to DMN) are sub-divided into two parcels (light and dark green) and vertices in the bottom right quadrant (task positive network, TPN) are sub-divided into four parcels which are distinct from those in the top right quadrant. While the spatial organization of these clusters in the square image appears random, when they are mapped back to the area on the cortex from which they were drawn, we see that the clustering result actually sub-parcellates the DMN and TPN regions. This result demonstrates the “non-local” nature of tNLM—smoothing is performed based on similarity in time series rather than spatial proximity. For this reason, partitioning of tNLM-filtered data based on a fully connected graph can identify groups of pixels with similar time-series in the original data rather than producing false parcels as a result of local mixing of signals across functional areas as seen with LB filtering. This edge preserving nature of tNLM also allows accurate sub-division of functional regions when clustering is performed with larger value of *K*, as seen in the cortical map in Fig 3(f).

We do not include results for unfiltered data for the simulation since they are qualitatively very similar to those obtained using tNLM for both *K* = 4 and *K* = 8. Since each quadrant was selected to have clearly distinct time series from all other quadrants, the N-cuts algorithm, even without smoothing, was able to reliably partition the data for *K* = 4. Further, the unfiltered data produced a very similar sub-parcellation for *K* = 8 to that shown for tNLM in Fig 3(f), which indicates that there is evidence for these sub-parcellations in the data and these results are not an artifact of the nonlinear tNLM smoothing. As we show below, this similarity between tNLM and unfiltered data does not occur when N-cuts is applied to *in vivo* over the full cerebral cortex.

### Qualitative evaluation of cortical networks

**Fig 4:**
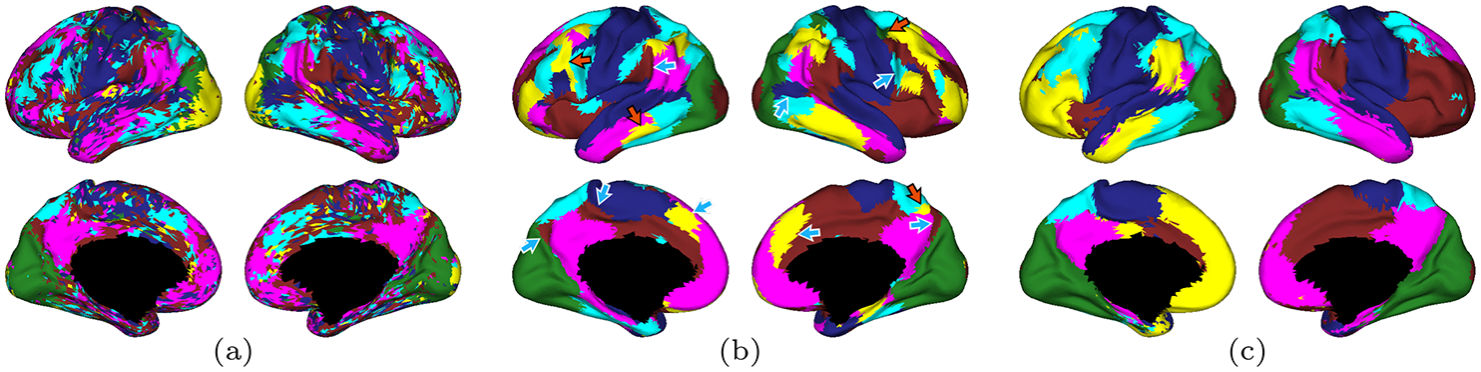
Cortical parcellations using N-cuts on a fully connected cortical surface graph for a single subject to partition cortex into *K* = 6 networks with (a) unfiltered data, (b) LB filtering (t = 4), and (c) tNLM filtering (h = 0.72). In each case a distinct color represents one of the *K* = 6 networks. Arrows in (b) illustrate regions lying between two large parcels that are classified as a separate network and appear similar to the false regions resulting from linear smoothing shown in the simulation in Fig 3(b).

Fig 4 shows the result of N-cut clustering with six classes for a single subject. Clusters obtained from the original unfiltered *in vivo* HCP data, Fig 4(a), yields default mode (pink), visual (yellow/green), and somatomotor (dark blue) networks. However, the clusters are noisy and disjointed. In contrast, tNLM smoothed data shows networks with large contiguous regions, Fig 4(c). In addition to the networks identified in the original data we can also identify the visual system (dark green) and and the cingulo-opercular (dark red) networks. We can also identify the fronto-parietal network, often described as anti-correlated to the default mode network (light blue), which includes the frontal eye field, left middle frontal gyrus, superior parietal lobule, and the lateral-posterior regions of the temporal lobe [32–36]. It is also noticeable that the DMN seems to be sub-divided in two clusters (in yellow and pink) in the tNLM parcellation. At smaller values of *K*, we do expect both hemispheres of the DMN to be grouped into a single label, as seen in tNLM N-cuts clustering with four classes, Fig C in S1 Text. With progressive increases of N-cut classes, *K*, we see the network systems continue to subdivide.

LB filtering, Fig 4(b), produces similar networks to those identified by tNLM, however some of the networks separate into a larger number of non-contiguous parcels, which appear as patches of small parcels throughout the cortex. Notice in the lateral surface of the frontal lobe, we see a mix of smaller patches of the yellow, pink, light blue, and dark blue labels which are isolated away from the large, spatially contiguous portion of each label. We also see that the inferior frontal gyrus (part of the language network) is fragmented into five different labels. At a coarse parcellation of the cortex into only six labels, we do not expect this system to be sub-divided. In contrast, tNLM seems to preserve the area as a part of larger cluster. Further, it can be also be noticed that several regions between two large networks are classified as a separate network as indicated by the arrows. A greater number of patchy regions in the LB result, particularly near boundaries of known networks, is consistent with the formation of additional false parcels in the simulation study in the previous section.

**Fig 5:**
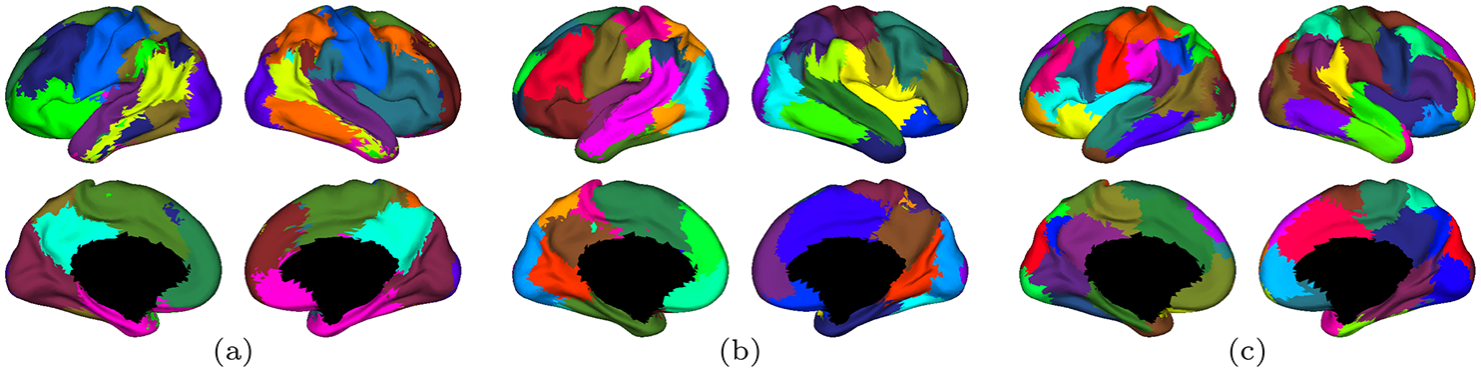
Cortical parcellation N-cuts applied to tNLM filtered (h = 0.72) data for the same subject as in Fig 4 for (a) *K* = 15, (b) *K* = 30 and (c) *K* = 60 clusters. See Fig C in S1 Text for equivalent images for unfiltered and LB filtered data.

As the number of subgraphs increases with (a) *K* = 15, (b) *K* = 30, and (c) *K* = 60, we notice that boundaries are frequently preserved and regions are sub-divided with tNLM filtering (Fig 5) while the equivalent results for unfiltered data become increasingly noisy, and LB filtering continues to produce apparently spurious regions which appear as patches of small clusters around boundaries of larger networks (Fig C in S1 Text). For example, for tNLM results, the somatosensory and motor cortices are initially identified as a single network (blue) for *K* = 15 classes (Fig 5(a)). When the number of classes is doubled, this area sub-divides into the right upper (violet), and left upper (pink), and the lower (brown) somatomotor cortices (Fig 5(b)). Increasing the number of classes to 60, the right lower somatomotor cortex (red) separates from the left hemisphere, and further divides into the ventral premotor cortex (blue) and the ventral motor cortex (dark red) (Fig 5(c)). Similar patterns of progressive subdivision can be observed in other primary networks including the visual network.

**Fig 6:**
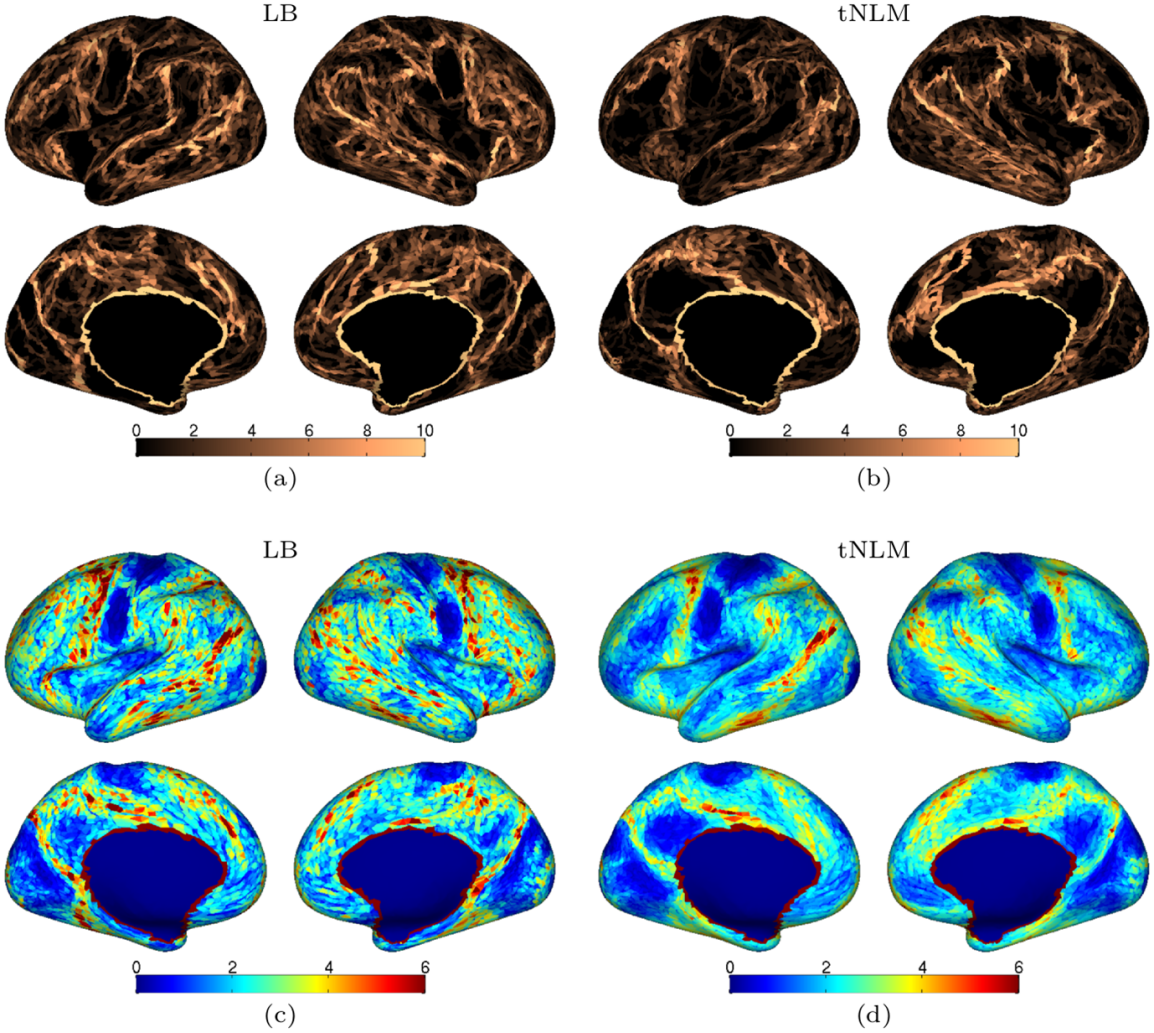
Cortical map of the cumulative boundaries of N-cut parcellations over fifteen different values of *K* in a single subject with (a) LB (*t* = 4) and (b) tNLM (*h* = 0.72) filtering. The population average cumulative boundary map across the 40 subjects are also shown with (c) LB (*t* = 4) and (d) tNLM (*h* = 0.72) filtering. The value at each triangle represents total number of times that triangle was a identified as a boundary triangle across fifteen different clustering results (*K* = 2, 3, 4, 5, 6, 7, 8, 9, 10, 15, 30, 40, 50, 60, 80). The boundary maps are thresholded at an upper boundary count of 10 for single subject and 6 for the population average.

In Fig 6 we illustrate how the boundaries of the clusters vary across several parcellations with different number of classes in both tNLM and LB. Fig 6(a) and 6(b) shows cumulative boundaries for a single subject over different numbers of classes. The tNLM results show consistent boundaries delineating the ventro-medial prefrontal cortex, posterio-medial cortex (PMC) and the visual cortex. The LB results are quite similar, however, we notice a larger number edges running through the primary network regions. For example, several edges are seen in the interior of PMC and similarly some edges are seen in the interior of the medial side of visual cortex running parallel to the boundary between visual cortex and PMC on both hemispheres. When cumulative edges are averaged across the population of 40 subjects, Fig 6(c) and 6(d), we see boundaries occurring more consistently across different values of *K* with tNLM filtering than with LB filtering. In both cases the upper and lower sensorimotor areas are consistently identified, as evidenced through the absence of boundaries in these regions. However, tNLM shows clearer boundaries than LB, particularly for the PMC, the visual cortex and the ventrolateral prefrontal cortex. Higher order association cortices also clearly show marked internal consistency with tNLM. LB shows boundaries spread across frontal and lateral posterior temporal-occipital region, again, possibly reflecting the introduction of false parcels near boundaries between functional areas as illustrated earlier in the simulation study.

Additional results showing the distribution of the number and size of clusters in LB and tNLM N-cuts parcellations are included as Figs D–G in S1 Text. These results support the general observation that LB results tend to produce more, smaller contiguous clusters than tNLM.

### Quantitative comparison with task fMRI labels

**Fig 7:**
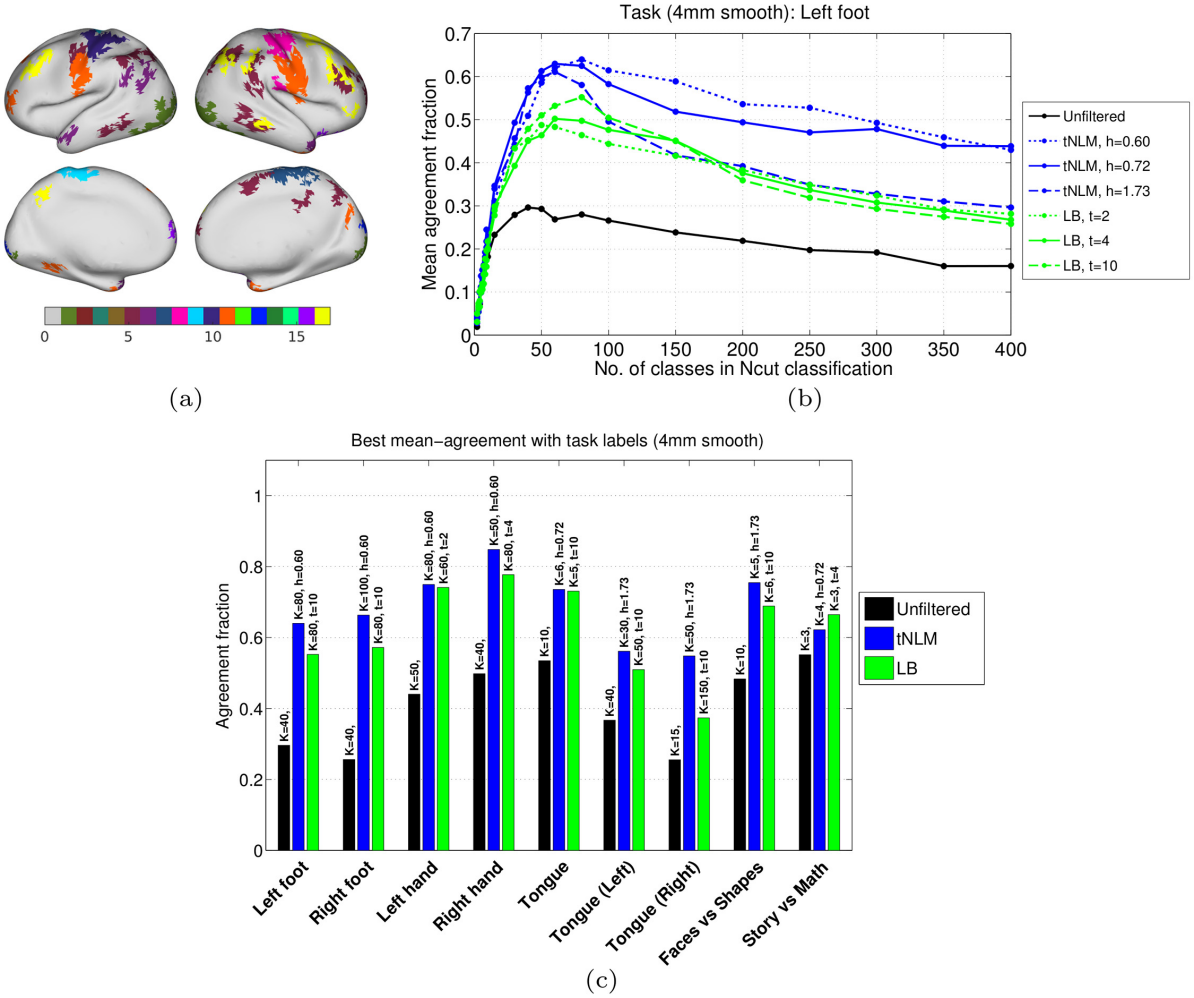
Quantitative comparison with task labels. (a) An example of task labels for a single subject obtained from 4mm smoothed task fMRI data (see Methods section for task-pair for each label-ID) (b) Mean agreement fraction, across 40 subjects × 4 sessions, of an example task label (Left foot, motor task) with N-cuts parcellations obtained using unfiltered, LB filtered and tNLM filtered rfMRI data. See Fig K in S1 Text for corresponding plots for all task labels. (c) Best performance of different filtering approaches across different task labels. For each task and each filtering approach, we select the parameters which achieves the highest mean agreement fraction. The grouped bar plot shows the highest mean agreement fraction and the text on top shows the corresponding parameters.

We quantify the quality of parcellations obtained with different filtering approaches by computing the agreement of rfMRI parcellation with task labels for each subject An example of task labels for a single subject is shown in Fig 7(a). For each subject, we investigate the agreement of each task label across several rfMRI parcellations obtained with different numbers of classes (2 ≤ *K* ≤ 400) and with different filtering approaches: unfiltered, LB filtering (*t* = 2, 4, 10) and tNLM filtering (*h* = 0.60, 0.72, 1.73). Fig 7(b) shows the mean agreement fraction of an example task label, left foot motor task, with clustering results across the population. Similar plots for other task labels are included in Figs I–L in S1 Text. For the tongue motor task, we also studied the performances by subdivided the tongue region into two regions, one on each hemispheres, because N-cuts clustering frequently sub-divided the clusters across hemispheres for *K* > 15.

The mean agreement fraction varies substantially with number of classes *K* for all filtering approaches and all task labels. This behavior indicates a potential limitation of the N-cuts approach in that a single value of *K* will not optimally segment all regions, which presents a challenge in selecting an appropriate value of *K* when using this method in practice. However, our purpose here is to evaluate the relative impact of different forms of smoothing rather than advocate a specific parcellation approach. To compare performance, we therefore selected the ‘best’ parcellation for each task and each filtering approach (in terms of parameters *K*, *t* and *h*) as that which achieves the largest mean agreement across the population for that task. We then compare the performance of different filtering approaches for their quantitatively ‘best’ parcellation for each task label in Fig 7(c). This comparison shows a substantial improvement in performance over unfiltered data with both LB and tNLM but overall, tNLM filtering achieves a larger mean agreement fraction. More complete overall results for different levels of smoothing of the task data are shown in Fig I in S1 Text with detailed results for individual tasks shown in Figs J–L in S1 Text.

We also performed a non-parametric test to examine the statistical differences between the ‘best’ parcellations of LB and tNLM filtering for each task label, the results of which are presented in Table 1. We used the Wilcoxon signed-rank paired test [37] with a null hypothesis of no subject-wise difference in ‘best’ performances between LB and tNLM filtering. The alternate hypotheses for the signed-rank paired tests (*i.e.* which of tNLM or LB performs better) were decided by the value of the population median of agreement fractions and are reported in Table 1 for each task-pair.

**Table 1:** Statistical tests for improved agreement with task labels: Table of (uncorrected) p-values for signed-rank test for ‘best’ performance of LB and tNLM filtering (see text for detailed description). For each task label, the best performance parameters for both filtering approaches are reported in Fig 7(c). The agreement fractions across population, computed with these filtering parameters, are used as the performance metric for the tests. The alternate hypothesis “tNLM>LB” means that the median agreement fraction of the tNLM approach is greater than the median agreement fraction of the LB approach; and similarly for “LB > tNLM”.

Task-pair (ID)	Alternate hypothesis	Signed-rank p-values
Left foot (7)	tNLM > LB	7.588 × 10^−9^
Right foot (9)	tNLM > LB	1.7304 × 10^−7^
Right hand (10)	tNLM > LB	2.7763 × 10^−13^
Tongue (Left)	tNLM > LB	3.4968 × 10^−6^
Tongue (Right)	tNLM > LB	6.995 × 10^−20^
Faces vs. Shapes (1)	tNLM > LB	1.8794 × 10^−9^
Tongue (11)	LB > tNLM	1.6967 × 10^−3^
Left hand (8)	LB > tNLM	3.9957 × 10^−3^
Story vs. Math (6)	LB > tNLM	9.4594 × 10^−9^

### Quantitative test-retest reliability

We also investigated the consistency of rfMRI parcellations across different scan sessions of the same subject. For each subject, a total of four independent fMRI data were collected over two-days (two 15 minutes rfMRI runs each day), as per HCP fMRI protocol [6, 24, 38]. We computed the concordance between all 6 possible pairings of the parcellation results for each of the four 15 min sessions. Concordance is a measure of consistency between parcellations which measures the fraction of vertices which agree between the parcellations and is described in details in section C in S1 Text. The concordance measure was computed over all 40 subjects for each method and several parameter settings.

**Fig 8:**
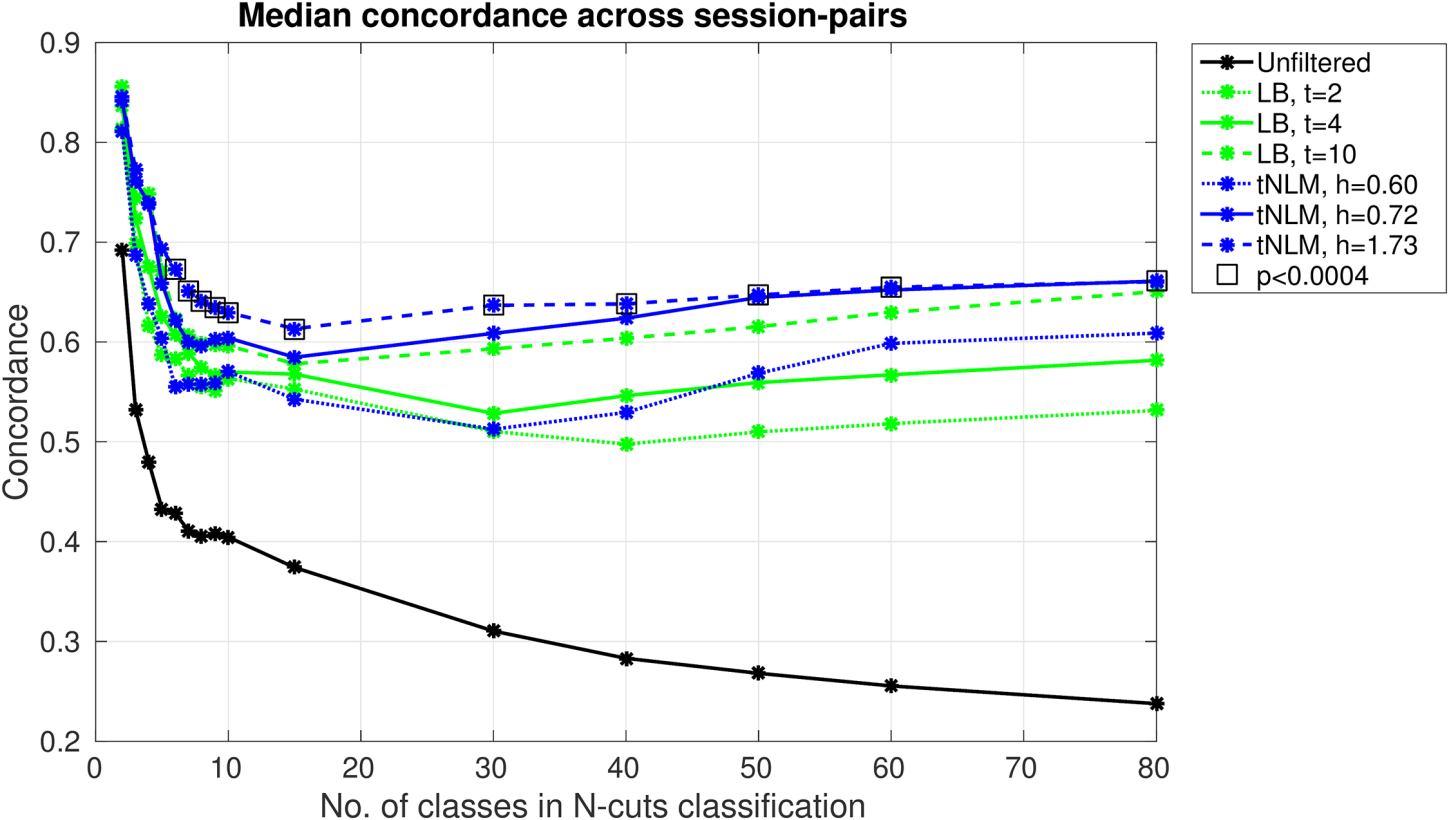
Test-retest reliability. Median concordance of parcellation results over the pairs of rfMRI sessions (40 subject × 6 session pairs) as a function of the number of cuts, *K* = 2 to 80, for different filtering approaches. Square boxes indicate significant differences (uncorrected p-value < 0.0004) between best of tNLM and LB median concordance values, as tested with Mann-Whitney U (rank-sum) test.

In Fig 8 we show within-subject agreement over all six possible pairs of parcellations from the four 15-min rfMRI sessions per subject. We plot the median concordance, over the six pairs per subject and the 40 subjects, as a function of the number of cuts, *K*, for three different smoothing parameters for LB and tNLM. Non-parametric Mann-Whitney U (rank-sum) tests for significant differences (uncorrected) between median performances of tNLM and LB were also performed and the square boxes indicate values of p<0.0004. These results show concordance for LB and tNLM roughly decreases until *K* = 10, and then remains relatively stable but with a slow increases as *K* increases to 80. The unfiltered data results are far worse than either LB or tNLM. Over the range from *K* = 6 to 80, tNLM with *h* = 0.72 and *h* = 1.73 consistently outperform the other methods and settings. Qualitatively, tNLM with *h* = 0.72 and LB with *t* = 4 produced the best apparent results, and there is a clear difference in performance here between these two over the same range (*K* = 6 to 80). While it is initially surprising that the concordance improves with *K* (this trend continues for *K* > 80 with LB ultimately given significantly larger concordance than tNLM for *K* > 130, see Fig O in S1 Text, we believe this is simply a result of the decreasing size of the clusters in each network with increasing *K* and the nature of the matching algorithm used to compute concordance. Consider the limiting case where *K* = *number of vertices*. Then each surface vertex forms a different parcel. In this case, the matching algorithm would achieve a perfect match and 100% consistency. Figs D–G in S1 Text show that cluster-sizes of LB parcellations are substantially smaller than tNLM, especially for *K* > 130. Cluster-size distribution in Fig D in S1 Text shows that tNLM has several clusters which are substantially larger than LB for *K* ≥ 100. The distribution of cluster-size in Fig E in S1 Text shows this effect more clearly, where we see that tNLM results have substantially larger clusters as compared to LB across most of the cortex. Fig F in S1 Text shows that tNLM results seems to consistently have several large clusters as well as several small clusters. In comparison, LB results seems to uniformly divide the whole cortex in approximately equal size for large *K*, Figs D and F in S1 Text. Since the average size of the clusters in LB for a fixed *K* is substantially smaller than that for tNLM (Fig F in S1 Text) there is an increased chance of a (random) good match between pairs of parcellations. Figs P and Q in S1 Text illustrate the spatial distribution of concordance/disagreement in region boundaries for tNLM and LB as a function of *K*.

## Discussion

The tNLM filtering results shown in Fig 1 and the accompanying video, S1 Video, illustrate the qualitative impact of this form of nonlinear filtering on resting BOLD data. There are relatively few other examples showing real time rfMRI whole-brain activity in the form of either single frame images or movies in the literature. An early example from Vincent et al. shows real-time brain dynamics from data used to explore resting networks (https://youtu.be/VaQ66lDZ-08 and https://youtu.be/3nCBLw9Z-xU) [36, 39] but the dynamics do not clearly show coherent activity in regions forming the default mode and other networks. Another example from Raichle (http://www.nil.wustl.edu/labs/raichle/images/Restless_Brain/restless_brain_lat.html) also demonstrates dynamic behavior in resting fMRI similar to the tNLM video but at a slower rate [40]. Kundu et al. [41] have developed a method for denoising multi-echo fMRI data that distinguishes BOLD from non-BOLD signals based on echo-time dependence. The resulting denoised data show dynamic activation in DMN and other networks in real-time (https://youtu.be/D_UUfIF49Vc) similar to that shown in Figs 2 and 1. However this approach explicitly requires a multi-echo sequence and unlike tNLM cannot be applied to standard fMRI protocols. The dynamics shown in the tNLM movie also share similarities with those in Zalesky et al. [42] who explore the HCP resting data data using dynamic regional network efficiency measures, computed from time-resolved connectivity estimates, to produce movies of brain dynamics. Their results show “a consistent set of functional connections (with) pronounced fluctuations in their strength over time”. The authors also note spontaneous increases in spatially distributed regions over brief intervals, observations that can be also be made from the tNLM movie, S1 Video.

The effect of LB filtering relative to tNLM is shown in the S3 Video and Fig 2. Because LB filtering does not account for temporal correlations when performing spatial averaging, the method will tend to blur boundaries between distinct functional regions. As a result, as activity in one region increases and that in an adjacent one decreases, the effect of LB smoothing produces an apparent boundary or wave moving over time from one region to the other. This behavior is apparent in the LB movie (S3 Video). In contrast, with tNLM only vertices with similar time series are averaged to denoise the data. Therefore when two adjacent regions have distinct time series, they should not be blurred through filtering and this wavelike effect does not occur. Qualitatively, there are clearer boundaries in the tNLM images relative to LB, although it is also evident that even within regions corresponding to a single network, activation is not always synchronous. Overall, the impression given in the tNLM movie, S1 Video, is that of irregular, burst like activity, similar to what might be expected during a period of undirected mind-wandering [43, 44]. This ability to denoise fMRI data without excessive blurring across functional boundaries may make tNLM useful in exploratory studies of dynamic fMRI data involving resting, movie watching and other non-repetitive paradigms.

In addition to Gaussian filtering several other post-processing approaches have been explored for denoising the fMRI data [6, 45–48]. These approaches include bilateral filtering [49], ICA (Independent Component Analysis) based filtering [50–52], Wavelet-based denoising [53, 54] and Markov Random Field (MRF) based filtering [45]. MRF filtering uses a combination of spatial and temporal similarity measures to perform nonlinear smoothing based only on local (in time and space) data which, unlike tNLM, will result in a time-varying effective smoothing kernel. ICA filtering does make use of ‘non-local’ similarity but does not perform the weighted spatial averaging which tNLM uses to produce the functional-boundary preserving results shown here. While each of these non-Gaussian methods [45–54] has been shown to be effective for particular applications, Gaussian filtering remains the most widely used approach among fMRI studies and is frequently effective [6, 55–57]. Hence, all comparisons in this paper are with LB filtering, which is a generalization of Gaussian filtering on cortical surfaces. Further, some of these non-Gaussian filtering approaches can also be used simultaneously to combine their respective advantages. For example, all the data presented in this paper were filtered for non-neuronal signals using an ICA-based approach [6, 24] before LB or tNLM was applied.

Denoising with tNLM is also potentially attractive as a precursor to network identification or parcellation. The images in Fig 4 illustrate the beneficial impact of filtering prior to clustering on the ability of N-cuts parcellation to form larger piece-wise contiguous regions. However, LB filtering appears to produce a larger number of spurious clusters, as indicated in Fig 4(b) and 4(e). An example of how and why these spurious clusters may be formed as a result of LB smoothing is shown in the simulation example, Fig 3. The presence of spurious clusters around boundaries of large networks can also be appreciated by comparing Figs F and G in S1 Text. These figures show the average cluster-size as a function of location on the cortical surface before and after breaking networks into contiguous parcels. For the case *K* = 100, for example, the average contiguous cluster sizes are quite similar in the tNLM parcellation, while LB results show a band of small clusters (the yellow regions) along the boundary of the visual cortex. These are consistent with the spurious boundary regons produced in the simulation, Fig 3. This simulation result, and the corroborating evidence from *in vivo* data, indicates that (regardless of the merits of tNLM filtering) care should be taken when using linear smoothing in combination with parcellation methods based on pairwise correlations to ensure that parcels are not produced solely as an artifact of smoothing.

While all examples shown here are restricted to cortex, tNLM filtering can also be applied to volumetric or grayordinate [7] representations of the data. Similarly, tNLM could be applied as a denoising tool in event-related functional MRI studies which may result in improved resolution of focal activation relative to methods based on conventional isotropic linear smoothing. The tNLM method could also be extended to include a spatial component, so that the weighted average depends on a combination of temporal and spatial similarity. It would also be interesting to explore a dynamic version in which the similarity measure is computed of restricted time-window, rather than the entire time-course as was the case in the results presented above.

We illustrated the potential utility of tNLM through cortical parcellation studies based on N-cuts spectral graph partitioning. Our approach parcellates a single subject using a fully connected graph with edge strengths based on pairwise correlations between the time series on the surface elements. Most previous applications of graph-cuts in brain parcellation have used locally connected graphs to ensure spatially contiguous parcels [4, 5]. While this appears necessary for unfiltered data (Fig 4(a)), the denoising effect of tNLM or LB allows use of the fully connected graph while still producing a piece-wise contiguous parcellation (Fig 4(c)). This has the advantage of using all correlations for parcellation rather than a restricted subset, thus using more information.

Changes in parcellation as a function of the number of networks were investigated in Fig 5 for tNLM (with equivalent results in Fig C in S1 Text for LB and unfiltered data). A well known problem with N-cuts is that the algorithm tends to produce cuts of similar size if the graph (and its adjacency matrix) does not contain sufficient information to unambiguously support a single *K*-way partition [4]. We note that this does seem to be the case for relatively large numbers of networks (*K* ≥ 60) as shown in Fig 5. The plots of cluster-size distribution in Figs D and F in S1 Text also supports this view. As noted by Blumensath et al. [4], a hierarchical approach may produce superior results to N-cuts for these larger numbers of clusters.

To investigate consistency as a function of the number of networks, we averaged edge locations over multiple values of *K* in Fig 6. It is interesting that the resulting individual edge maps (Fig 6(a)) bear a strong resemblance to the group functional connectivity gradients shown in Fig 10 in [6].

Quantitative comparisons with functional task labels (and Brodmann areas in section D in S1 Text, Figs M and N) demonstrate that tNLM filtering achieves improvement over LB filtering across most tasks and Brodman area maps over a wide range of *K* values. It is also interesting to note that both filtering approaches show peak performance around similar number of classes *K*.

## Conclusion

The results shown above support the primary claim of this report: that temporal non-local means (tNLM) filtering is able to denoise resting fMRI data while also retaining spatial structure that reflects ongoing dynamic brain activity. Correlated variations in activity are directly visible in the tNLM movie of cortical activity, and appear to reflect the underlying dynamics of large-scale brain networks. While linear LB filtering produces smoothed results that also reveal dynamic brain activity, the fact that this form of smoothing does not consider temporal correlations will inevitably result in a blurring of functional boundaries. The simulation and experimental results presented above indicate that this can also lead to spurious results when LB filtering is applied prior to cortical parcellation. This ability of tNLM to help visualize real-time whole-brain networks may facilitate exploratory data analysis leading to new insights into the dynamics of spontaneous brain activity. Temporal NLM can also be used as a preprocessor for resting fMRI for exploration of dynamic brain networks and achieves significant improvement over traditional isotropic smoothing in quantitative comparisons.

## Supporting Information

S1 Text Supplemental Material. Appendices and additional detailed results.
(PDF)

S1 Video tNLM filtered rfMRI data. Movie of temporal non-local means (tNLM) filtered (*h* = 0.72) BOLD signal intensities on the cortical surface for a subject from HCP dataset, played back at a real-time rate. The time series associated with each cortical vertex were normalized to zero mean and unit variance. The signal intensities are visualized on a smoothed cortical surface using a colormap with transitions from blue (negative) to white (zero) to red (positive). Movie Parameters: duration of 2.5 minute (200 rfMRI samples), 10 frames per second, 1 second in movies equals 1 second in real time.
(MP4)

S2 Video Unfiltered rfMRI data. Movie of unfiltered (minimally processed using HCP pipeline) BOLD signal intensities on the cortical surface for the same subject as S1 Video, played back at a real-time rate. The signal intensities are visualized on a smoothed cortical surface using a colormap with transitions from blue (negative) to white (zero) to red (positive). Movie Parameters: duration of 2.5 minute (200 rfMRI samples), 10 frames per second, 1 second in movies equals 1 second in real time.
(MP4)

S3 Video LB filtered rfMRI data. Movie of Laplace-Beltrami filtered (*t* = 4) BOLD signal intensities on the cortical surface for the same subject as S1 Video, played back at a real-time rate. The signal intensities are visualized on a smoothed cortical surface using a colormap with transitions from blue (negative) to white (zero) to red (positive). Movie Parameters: duration of 2.5 minute (200 rfMRI samples), 10 frames per second, 1 second in movies equals 1 second in real time.
(MP4)
