# Supplementary material for: Temporal Non-Local Means Filtering Reveals Real-Time Whole-Brain Cortical Interactions in Resting fMRI
Source: PLoS One. 2016 Jul 8;11(7):e0158504. doi: 10.1371/journal.pone.0158504 (PMC4938391; doi:10.1371/journal.pone.0158504)
Supplement: S1 Text — Appendices and additional detailed results. (PDF) [file pone.0158504.s001.pdf]

Supplemental material: Non-local means filtering reveals real-time whole-brain cortical interactions in resting fMRI

A Dataset details

All the results presented in this report use data distributed by Human Connectome Project, WU-Minn Consortium (Principal Investigators: David Van Essen and Kamil Ugurbil; 1U54MH091657). We used the minimally preprocessed (ICA-FIX denoised) rfMRI data and pre-processed and analyzed task-fMRI data resampled onto individual cortical surfaces from 40 unrelated subjects [1, 2, 3]. The data is part of the 500 Subjects + MEG2 Data Release and was downloaded from http://www.humanconnectome.org/documentation/S500/. The IDs of the subject used in our study are: 100307, 101309, 103111, 105014, 116524, 117122, 118528, 118932, 120111, 122620, 123117, 123925, 126325, 128127, 129028, 130013, 130316, 131217, 131722, 133019, 133928, 135932, 149741, 151223, 151526, 151627, 159340, 162733, 163129, 176542, 188347, 190031, 192540, 196750, 198451, 201111, 221319, 499566, 751348, and 899885.

B Laplace-Beltrami filtering

Gaussian filtering is frequently used to isotropically smooth volumetric 3D data in functional and task based fMRI studies. Isotropic smoothing can also be performed on a 2D manifold such as the cortical surface. Because cortical surfaces have an intrinsic curvature that prevents them being mapped without distortion onto the plane, isotropic smoothing requires that curvature be taken into account. The Laplace-Beltrami (LB) operator, which generalizes the 2D Gaussian smoothing kernel from the plane to an arbitrary smooth manifold [4], can produce isotropic smoothing on cortex.

In this approach, a solution of the heat equation $\frac{\partial f(p,t)}{\partial t} = \Delta f(p,t)$ with initial condition $f(p,0) = Y(p)$ is used to smooth the data $Y(p)$, where Δ is the LB operator on the cortical manifold with spatial parameters p [5, 6, 7]. The parameter t represents the diffusion time for the heat equation. The degree of smoothing increases with t. The solution is given by [8, chap. 1]

$$f(p,t) = \int Y(q)\, K_t(p,q)\, \mathrm{d}q \tag{S.1}$$

where, $K_t(p,q)$ is the heat kernel which can be expressed in terms of ordered eigen-functions $\phi_0, \phi_1, \phi_2, \cdots$ and corresponding eigen-values $\lambda_0, \lambda_1, \lambda_2, \cdots$ of the Laplace-Beltrami operator Δ as [8, 6, 7]

$$K_t(p,q) = \sum_{n=0}^{\infty} \mathrm{e}^{-\lambda_n t} \phi_n(p)\, \phi_n(q). \tag{S.2}$$

The solution at time t can therefore be expressed as

$$\begin{aligned} f(p,t) &= \int Y(q) \sum_{n=0}^{\infty} \mathrm{e}^{-\lambda_n t} \phi_n(p)\, \phi_n(q)\, \mathrm{d}q \\ &= \sum_{n=0}^{\infty} \mathrm{e}^{-\lambda_n t} \phi_n(p) \int Y(q)\, \phi_n(q)\, \mathrm{d}q \end{aligned} \tag{S.3}$$

Eq. (S.3) can be expressed in discrete form as a square matrix of dimension equal to the number of vertices on the cortical surface using a truncated eigen-function expansion of the LB operator (we use the first 800 eigenfunctions

in all results with LB filtering in this paper). The level of smoothing of the final solution is parameterized by the time parameter t. The weights applied to neighboring vertices while filtering a particular vertex for different values of t can be intuitively interpreted as the smoothing kernel at the vertex. This approach allows efficient computation of filtered images as the eigen-decomposition needs to be computed only once irrespective of the number of rfMRI volumes or level of smoothing.

C Mapping and measures between parcellations

Assume we have two parcellations A and B for a set of vertices V: $A = \{A_i : i \in \mathbb{Z}_M\}$ parcellates V into M parcels where $\mathbb{Z}_M = \{1, 2, \cdots, M\}$ such that $\cup_i A_i = V$ and $\forall i \neq j$, $A_i \cap A_j = \varnothing$ and $B = \{B_i : i \in \mathbb{Z}_N\}$ parcellates V into N parcels where $\mathbb{Z}_N = \{1, 2, \cdots, N\}$ such that $\cup_i B_i = V$ and $\forall i \neq j$, $B_i \cap B_j = \varnothing$.

Label-wise agreement

Agreement is a label-wise measure of the fraction of vertices that agree between two parcellations A and B. Specifically agreement of parcel $A_i \in A$ with the corresponding parcel in B is given by

$$\text{Agreement}_{(A,B)}(A_i) = \frac{\left|A_i \cap B_{S(i)}\right|}{|A_i|} \tag{S.1}$$

where $|\cdot|$ represents the cardinality of a set and $S : \mathbb{Z}_M \mapsto \mathbb{Z}_N$ is a mapping of parcels in A to B as described later. The agreement measure ranges from 0 (no agreement) to 1 (perfect agreement).

Concordance: consistency between parcellations

Concordance is a global measure of consistency between two parcellations A and B. It is defined as the fraction of vertices between the two parcellations that agree:

$$\text{Concordance}(A, B) = \frac{\sum_{i \in \mathbb{Z}_M} \left|A_i \cap B_{S(i)}\right|}{\sum_{i \in \mathbb{Z}_M} |A_i|} \tag{S.2}$$

where $|\cdot|$ represents the cardinality of a set and $S : \mathbb{Z}_M \mapsto \mathbb{Z}_N$ is mapping of parcels in A to B as described next. The concordance measure ranges from 0 (no agreement) to 1 (perfect agreement).

Mapping between parcellations

Given the two parcellations A and B, we aim to match each parcel in A to a unique parcel in B. Let $g(A_i, B_j)$ be a measure of the goodness of the match of A_i to B_j such as the Dice coefficient or Jacard index. We want a map $\hat{S} : \mathbb{Z}_M \mapsto \mathbb{Z}_N$ such that we maximize the goodness of match across all parcels:

$$\hat{S} = \underset{S:\mathbb{Z}_M \mapsto \mathbb{Z}_N}{\arg\max} \left[\sum_{i \in \mathbb{Z}_M} g\left(A_i, B_{S(i)}\right)\right]. \tag{S.3}$$

The exact solution of eq. (S.3) is combinatorial and scales approximately as $n!$ where $n = \max(M, N)$).

We use an approximate solution of eq. (S.3) by noting its similarity to the famous stable matching problem [9]. Stable matching finds a match between elements of two sets of equal size when a preference order of matching is specified for each element. A match is stable if there does not exists a pair (a, b) in the match in which both a and b have higher preference elements which also prefer a and b respectively over their current match. We use the Gale-Shapley algorithm [9] after transforming our parcel mapping to a stable matching problem as described below. We will match each parcel in A to a unique parcel in B: In the language of the Gale-Shapley algorithm, elements in A are suitors and the elements in B are reviewers.

1. Compute an $M \times N$ matrix $\mathbf{G}$ such that the $(i, j)^{\text{th}}$ element $\mathbf{G}(i, j) = g(A_i, B_j)$.

2. The Gale-Shapley algorithm works with sets of the same size. Hence, we define an $n \times n$ matrix $\widetilde{\mathbf{G}}$ by appending an appropriate number of row or columns to $\mathbf{G}$. All the elements of appended rows/columns are set to $\delta = \min(\mathbf{G}) - \epsilon$, where ϵ is a small positive constant. This modifies our problem by adding dummy suitors/reviewers which can be easily ignored.

3. Next, we compute the preference order of each element from $\widetilde{\mathbf{G}}$. There are a total of n suitors and n reviewers. We define the preference order for each suitor by arranging the indices of the elements in the corresponding row in $\widetilde{\mathbf{G}}$ in the descending order of magnitude of their entries. Similarly, we define the preference order of each reviewer as the indices of the elements of the columns arranged in descending order of magnitude of their entries. If two elements have same preference, then we break the tie by randomly assigning a preference order.

4. We use the resulting preference order from the last step to find a stable match $\widetilde{S}$ using the Gale-Shapley algorithm [9]. The map $\widetilde{S} : \mathbb{Z}_n \mapsto \mathbb{Z}_n$ is modified to get $\hat{S} : \mathbb{Z}_M \mapsto \mathbb{Z}_N$ as follows: If $M = N$ then $\hat{S} \equiv \widetilde{S}$; If $M < N$ then all the appended dummy suitors are ignored in $\widetilde{S}$; If $M > N$ then all suitors which are matched to an appended dummy reviewer in $\widetilde{S}$ are modified to match to an empty set so that $B_{\hat{S}(i)} = \varnothing$.

The matching solution obtained by the above procedure is the suitor-optimal solution (in the sense of preference order). If a reviewer-optimal solution is required A and B should be swapped [9]. Further, the solution obtained is only an approximation of eq. (S.3) as the Gale-Shapley algorithm is blind to the absolute values of $g\left(A_i, B_{S(i)}\right)$ and only uses relative preference order, which may not always maximize the total cost. Nonetheless, in our experience, this approximate solution produces reasonable matching and is more computationally tractable than combinatorial approaches.

D Results

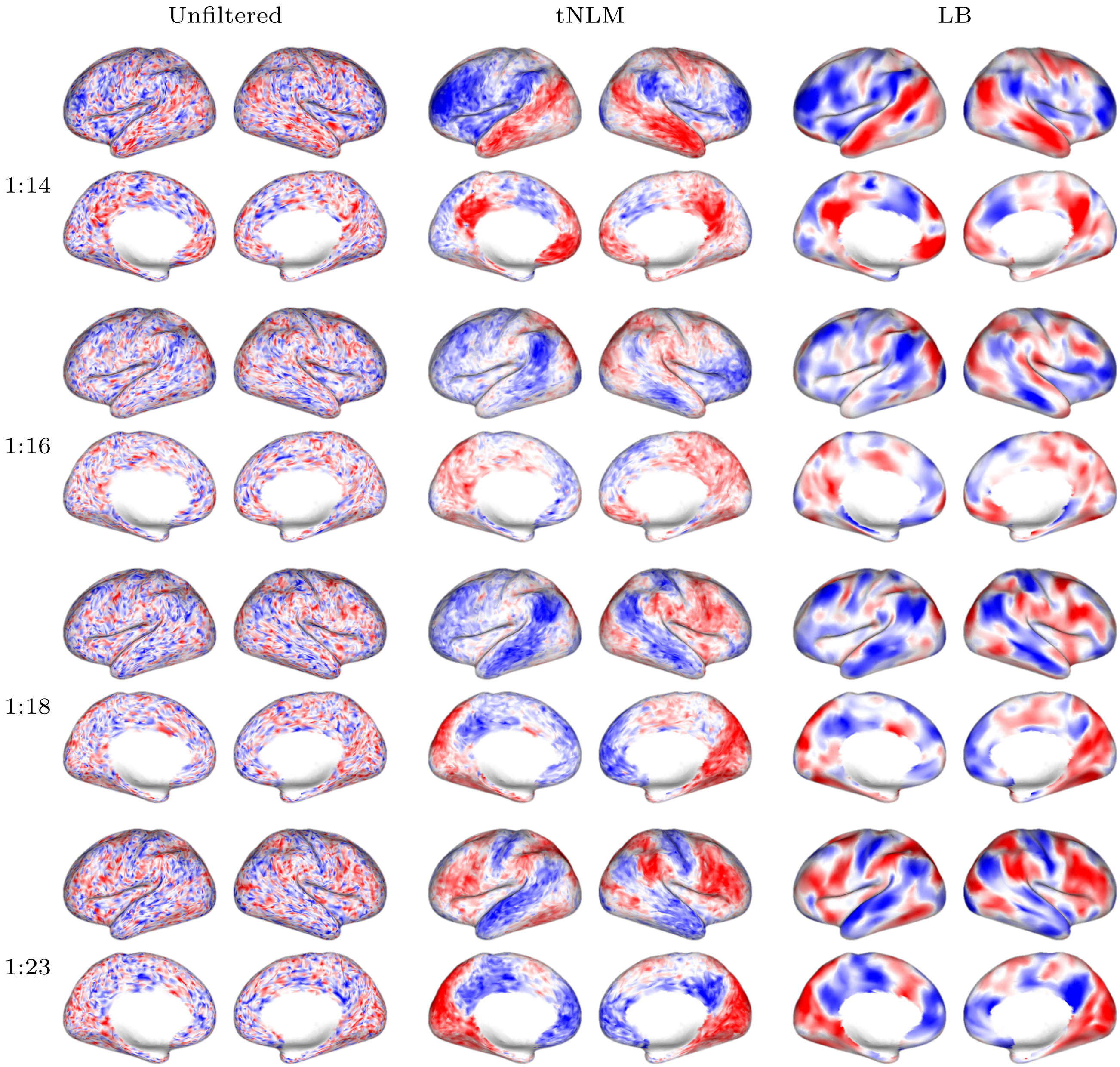

Figure A: Cortical distribution of the signal intensity of rfMRI data from a single subject at different time points (left) without filtering, (center) with tNLM filtering (h=0.72) and (right) with LB filtering (t=4). The video time points are the same as those in Fig. 2 in the paper and correspond to the times in the movies (S1, S2, and S3 videos) shown in left-most column. (Contd. to next page)

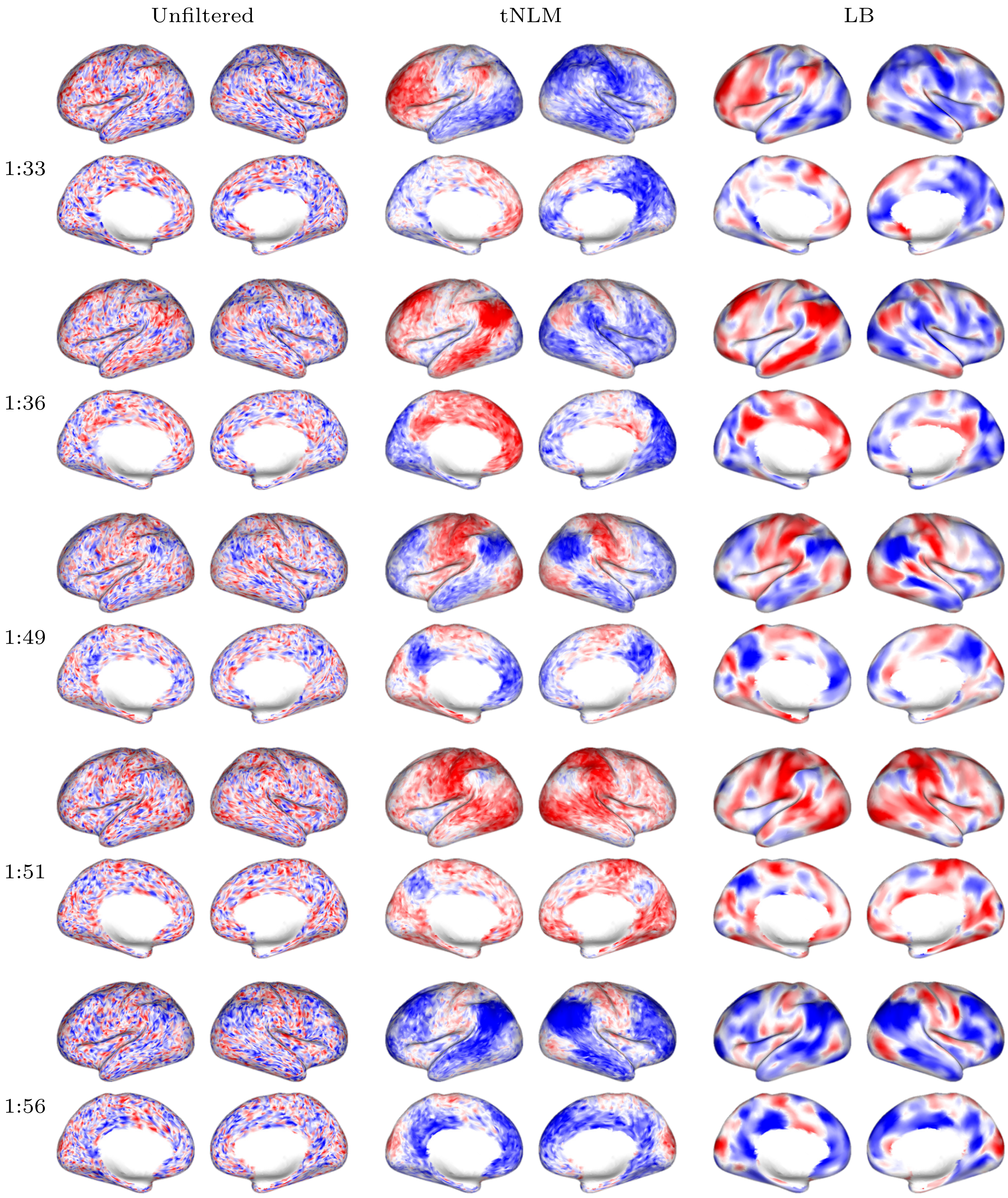

Figure A: (Contd. from previous page)

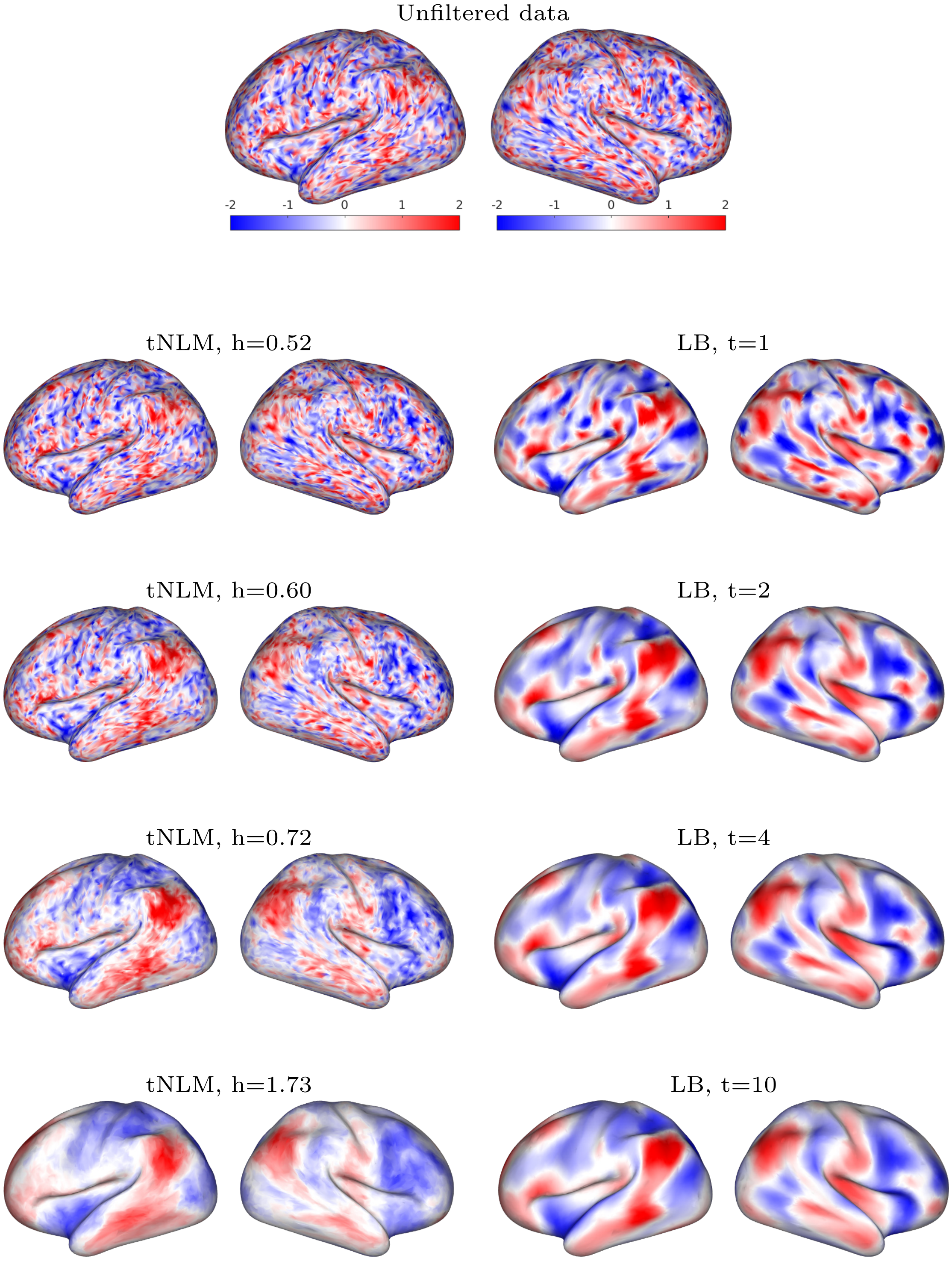

Figure B: Effect of tNLM and LB parameters on smoothing of the signal intensity of rfMRI data on the cortical surface. The original unsmoothed data is shown in the top image for one particular time instant. The result of filtering the original data with LB and tNLM ($D = 11$) with different parameters are shown in left and right columns. All the individual time series, unfiltered and filtered, were normalized to zero mean and unit variance before displaying as the signal intensity on the cortical surface.

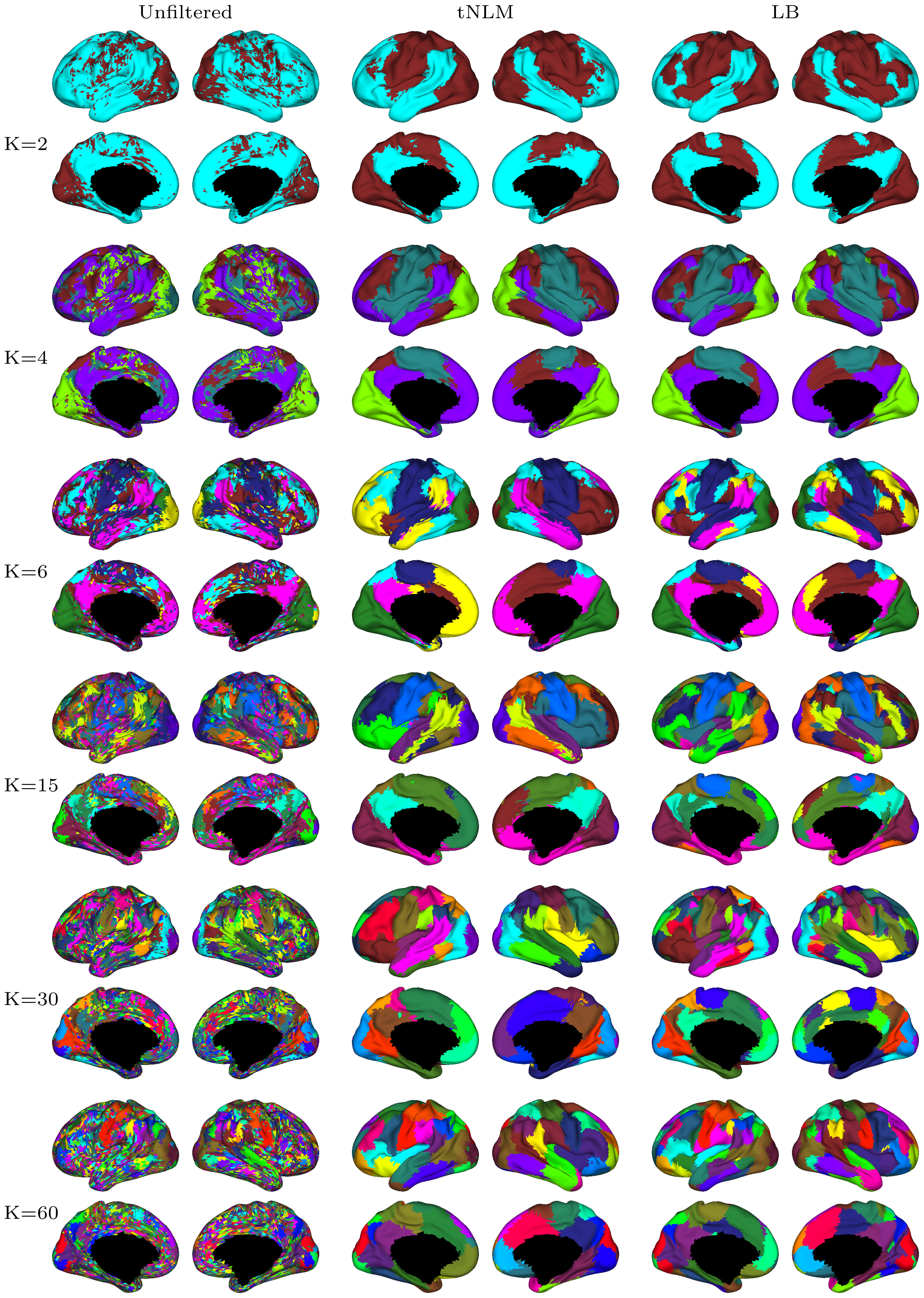

Figure C: Examples of cortical parcellation using full-graph with N-cuts obtained with unfiltered data, tNLM filtering (h=0.72) and LB filtering (t=4). The number of N-cuts classes (K) is shown in left-most column. Each N-cuts cluster is shown in a unique color such that the clustering result with K-classes will have K unique colors.

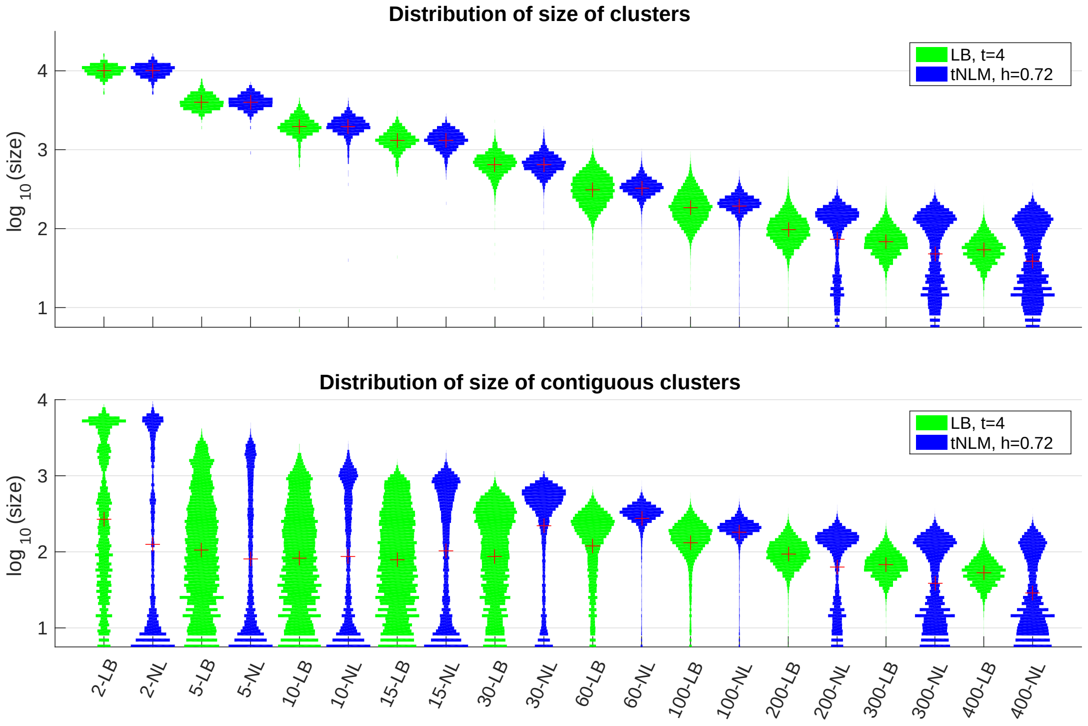

Figure D: Distribution of cluster-size over different values of K in N-cuts clustering with LB (t=4) and tNLM (h=0.72) smoothing across 40 subjects with 4 sessions each ($40 \times 4 = 160$ N-cuts clustering results). (Top) plot shows the cluster-size (vertex count) distribution for the clusters obtained by N-cuts. (Bottom) plot shows the cluster-size distribution of the *contiguous* clusters (obtained by breaking the N-cuts clusters into spatially contiguous parcels). The red + mark shows the mean size for each plot. Note that the cluster-size (y-axis) is reported on a logarithmic scale.

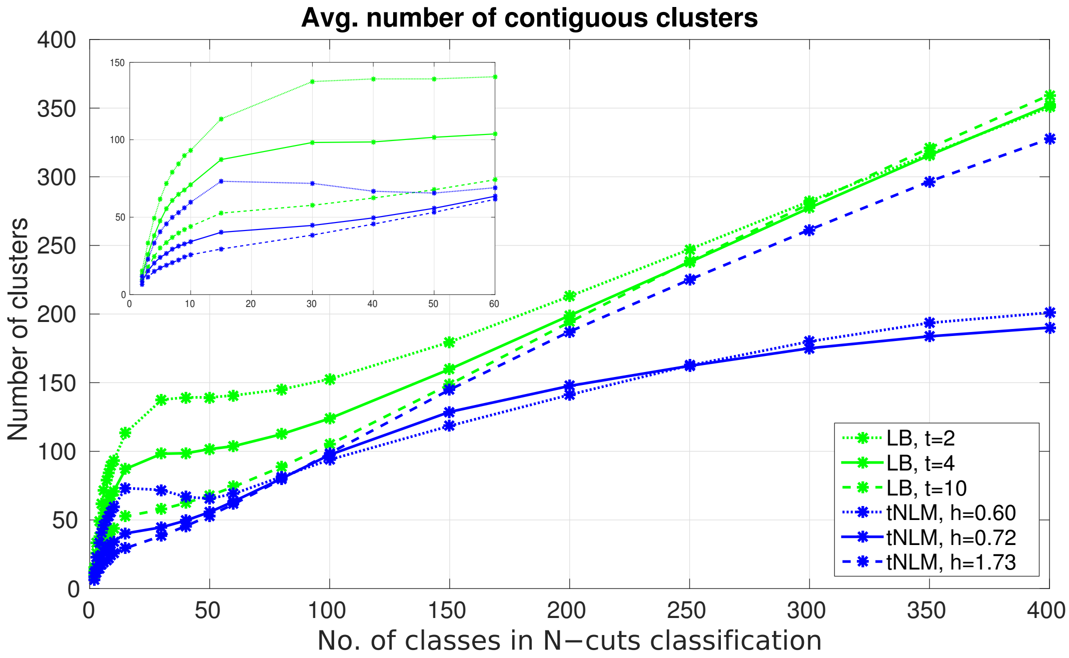

Figure E: Average number of *contiguous* parcels obtained using LB and tNLM filtering over different values of K in N-cuts clustering (the average is computed over 40 subjects $\times$ 4 sessions = 160 N-cuts clustering results). *contiguous* clusters are obtained by breaking N-cuts clusters into spatially contiguous parcels. The inset shows the zoomed-in view of the lower left corner. We have ignored all the contiguous parcels which have less than 20-vertices in the above count. Most of the ignored vertices lie in areas known for signal dropout in BOLD-EPI images (near sinus and ear-canal) as can be seen in fig. G (K=300,400).

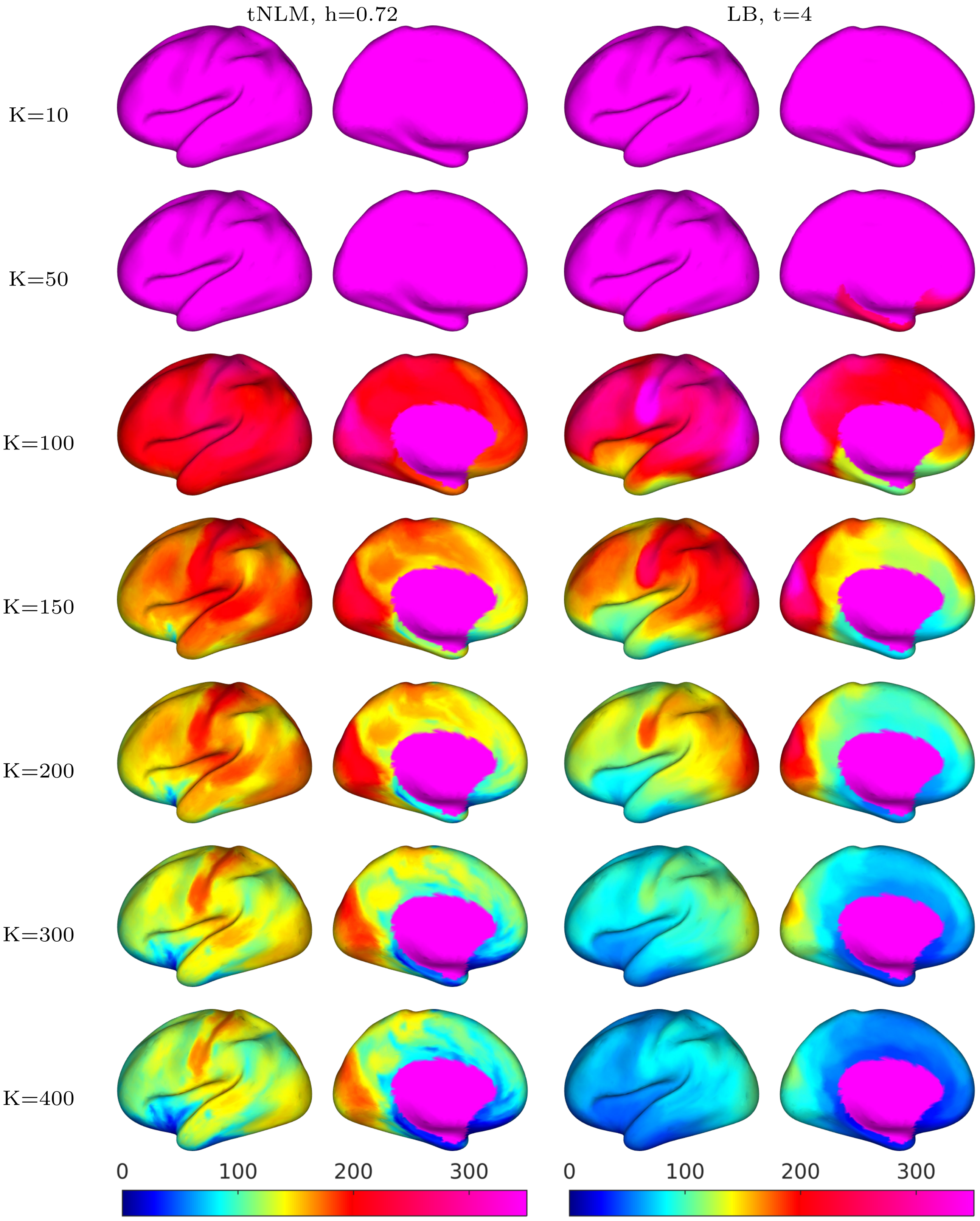

Figure F: Average size of N-cuts clusters, measured by average number of vertices in each cluster across $40 \times 4 = 160$ N-cuts clustering results (left) with tNLM filtering (h=0.72) and (right) with LB filtering (t=4). The value at each vertex represents the average size of the cluster to which that vertex belonged across all subjects/sessions. The map is thresholded at upper vertex count of 350.

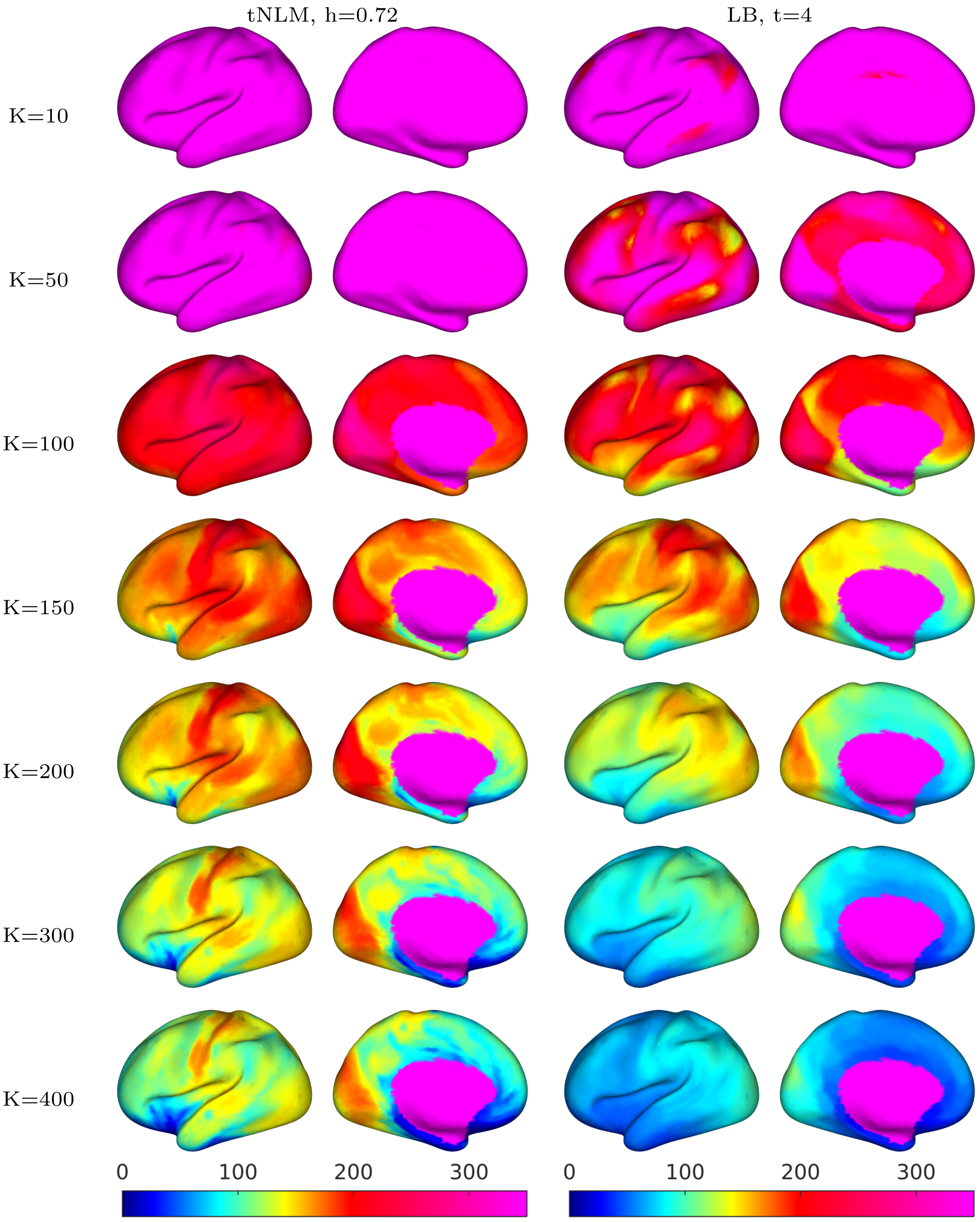

Figure G: Average size of *contiguous* parcels – the cluster-size computation is same as that in Fig. F except that the N-cuts clusters are first broken into *contiguous* parcels. The images use the same colorscale as Fig. F.

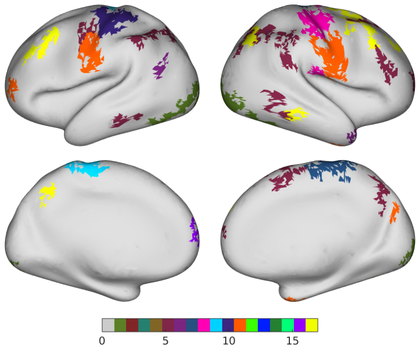

(a) 2mm Smoothed task fMRI

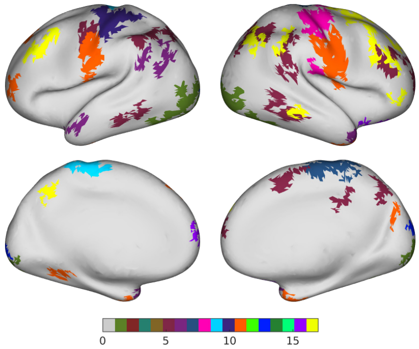

(b) 4mm Smoothed task fMRI

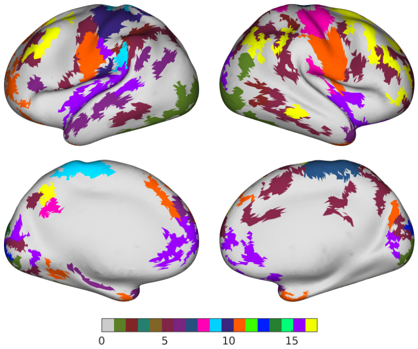

(c) 8mm Smoothed task fMRI

Task-pair	Label
Faces vs. Shapes	1
Shapes vs. Faces	2
Punish vs. Reward	3
Reward vs. punish	4
Math vs. Story	5
Story vs. Math	6
Left foot	7
Left hand	8
Right foot	9
Right hand	10
Tongue	11
Match vs. Rel.	12
Rel. vs. Match	13
Random vs. Tom	14
Tom vs. random	15
0-back vs. 2-back	16
2-back vs. 0-back	17

(d) Table of task-pair activation and label-IDs.

Figure H: An example of task labels for a single subject obtained by thresholding the task activation statistical maps at Z-score of 3.0 followed by ignoring all connected components with less than 40-vertices (see section 2.4.3 for more details). A total of 17 task-pair activation maps were obtained for each subject from HCP's processed data, which were computed by processing the task fMRI data with three different level of smoothing: (a) 2mm, (b) 4mm, and (c) 8 mm. The task-pairs corresponding to each task label is reported in (d). The 4mm smoothing result is also shown in Fig. 4 in the body of the paper and used for subsequent quantitative analysis in Fig. 10 and Table 1.

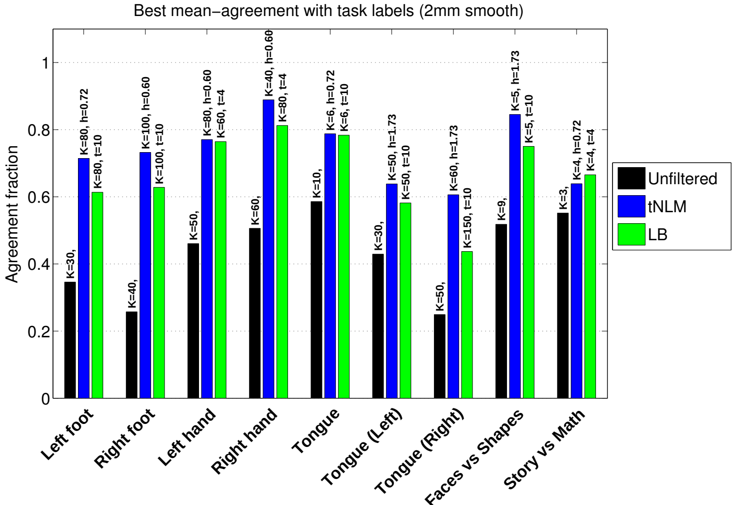

(a) 2mm Smoothed task fMRI

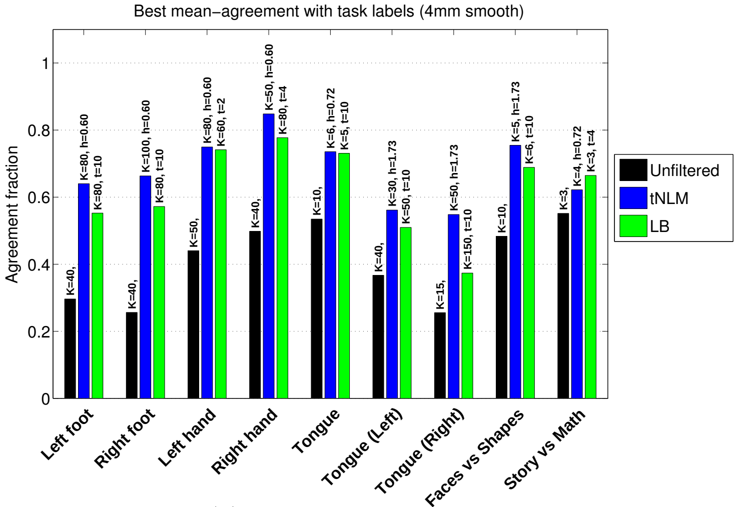

(b) 4mm Smoothed task fMRI

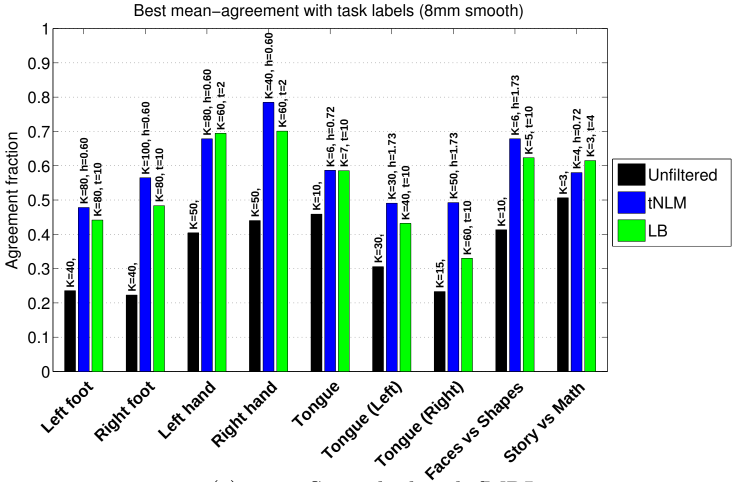

(c) 8mm Smoothed task fMRI

Figure I: Task-based evaluation: Best performance of different filtering approaches (no filtering, tNLM and LB) across different task labels, which were obtained by processing the task fMRI data with different levels of smoothing, shown in each sub-figure. For each task and each filtering approach, we select the parameters which achieves the highest mean agreement fraction (see Sec. 3.4 for more details). The grouped bar plot shows the highest mean agreement fraction and the text on top shows the corresponding parameters: h for tNLM and t for LB filtering and number of classes K in N-cuts. Figs. J – L shows detailed performance all filtering approaches across several parameters. Only a subset of these results are included in the body of the paper. Specifically, overall performance results for 4mm smoothing (Fig. 10(b)) and mean agreement fraction for left foot task and 4mm smoothing (Fig. 10(a)).

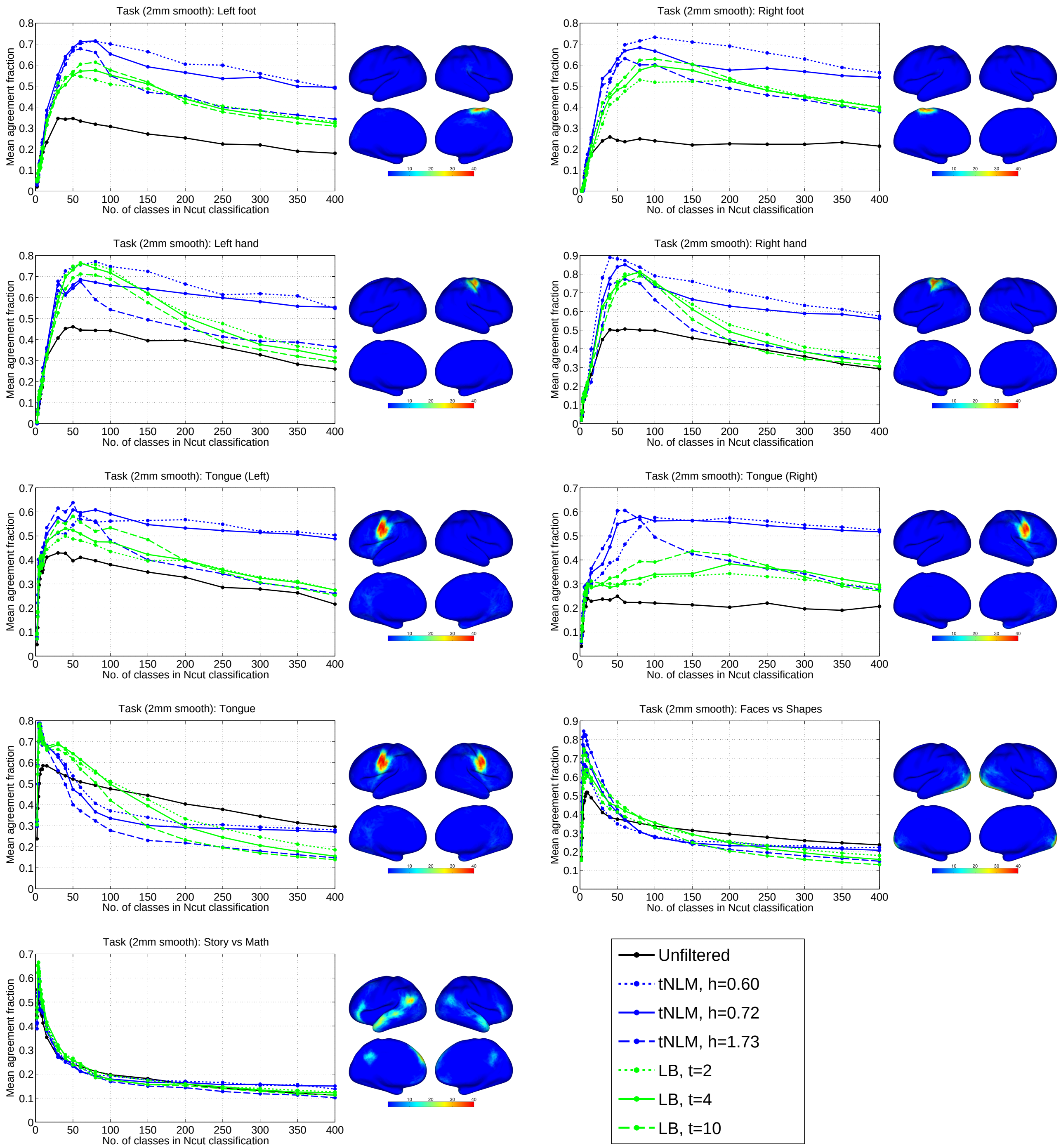

Figure J: Task-based evaluation: Detailed performance of all filtering approaches across several parameters for task-labels obtained by 2mm smoothing of task fMRI data. Line plots show the mean agreement of N-cuts clusters with 2mm-smoothed task labels across 40 subjects with 4 sessions each ($40 \times 4 = 160$ N-cuts clustering results). For each task, we also show the spatial histogram of the task label across the 40 subjects. Only seven tasks, shown above, could survive the statistical and contiguity thresholding (see section 2.4.3 for more details), and can also be seen in Fig. H. The tongue task was also studied after breaking the activation maps into two parts, each lying on one hemisphere of the brain.

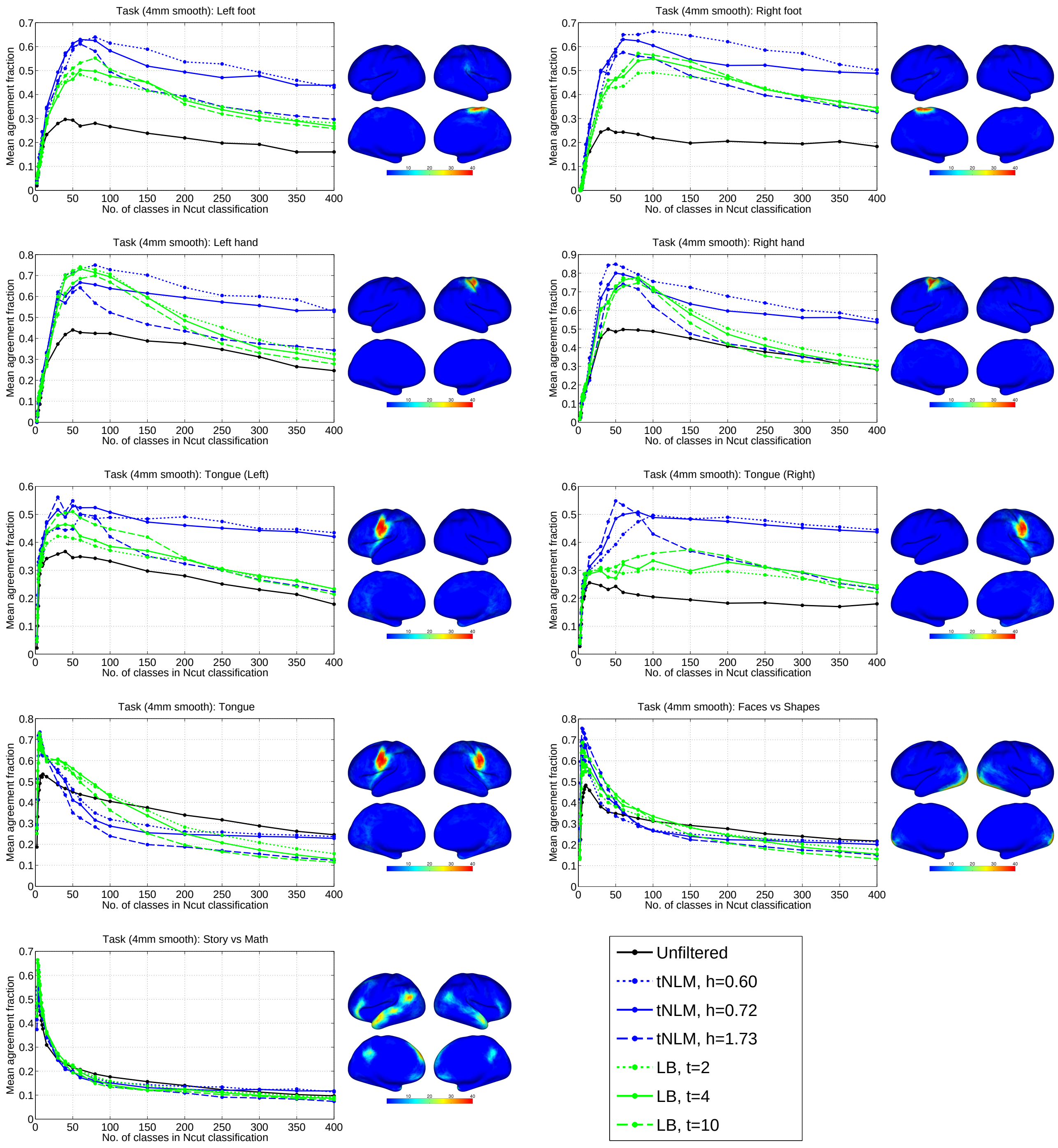

Figure K: Task-based evaluation: (Same as Fig. J, but with 4mm smoothing) Detailed performance of all filtering approaches across several parameters for task-labels obtained by 4mm smoothing of task fMRI data.

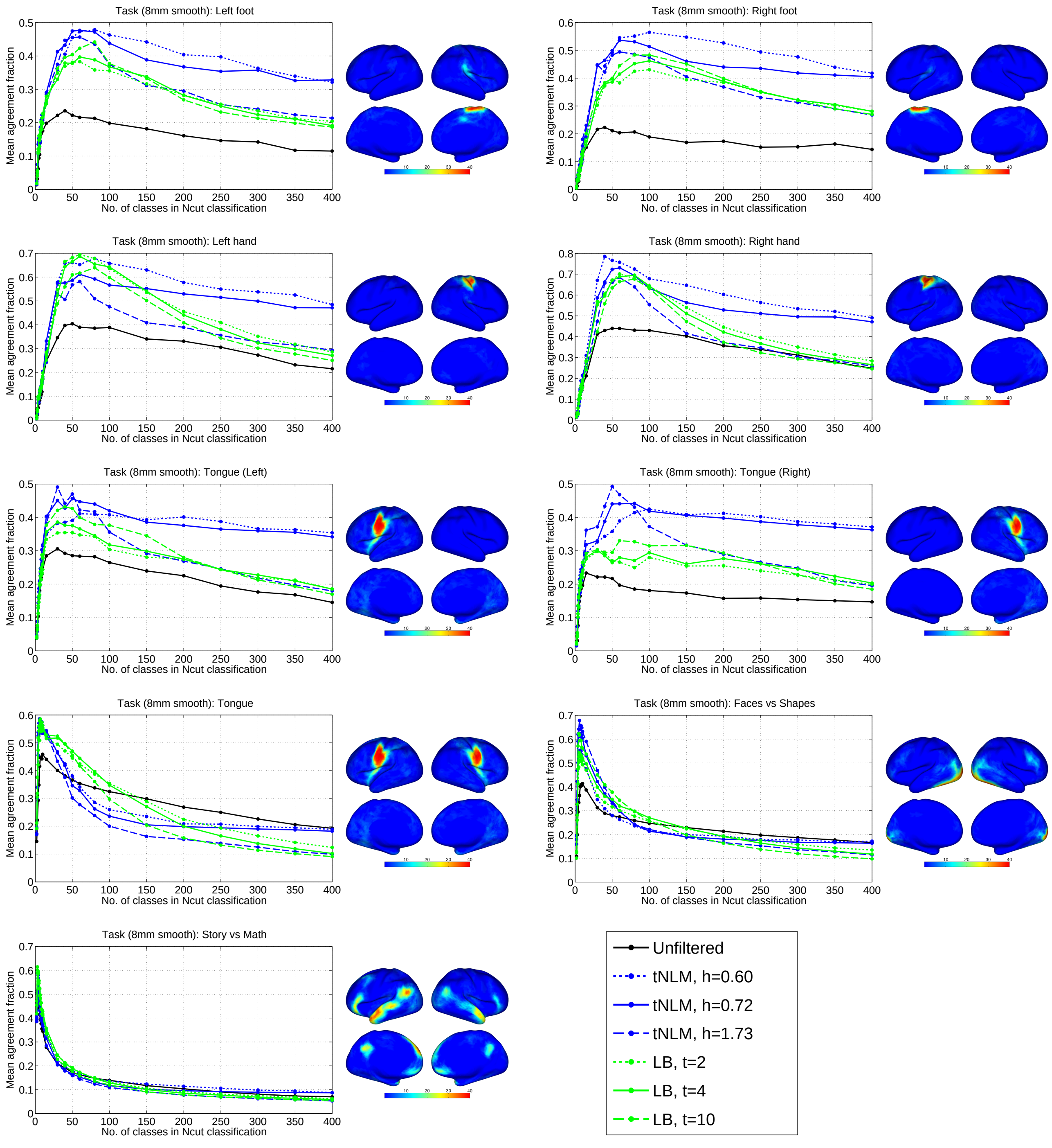

Figure L: Task-based evaluation: (Same as Fig. J, but with 8mm smoothing) Detailed performance of all filtering approaches across several parameters for task-labels obtained by 8mm smoothing of task fMRI data.

Quantitative comparison with probabilistic Brodmann areas (BAs)

Since, it is believed that the cytoarchitectonic areas reflect functional specialization in cerebral cortex [10], it is also interesting to study how the functional parcellations obtained from rfMRI data compare with probabilistic Brodmann areas (BA). Probabilistic BA were obtained using histology studies of 10 postmortem human brains and were transferred to the subject's 32K Conte-69 cortical mesh, which are available from HCP as discrete labels (after thresholding) for each subject [11, 12]. An example of the discrete Brodmann label map is shown in Fig. M(a). Similarly to our task based analysis, we computed label-wise agreement between BA labels and N-cuts parcellations to study the effect of different filtering approaches.

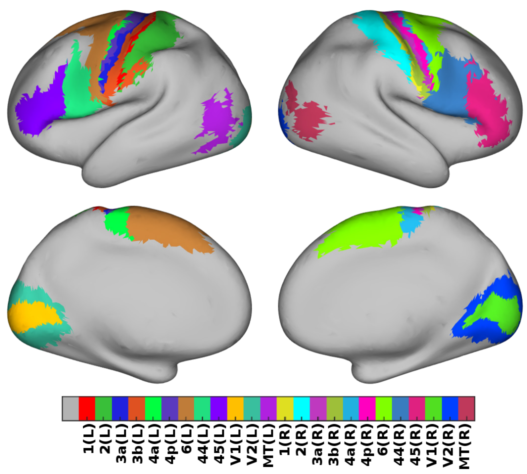

(a)

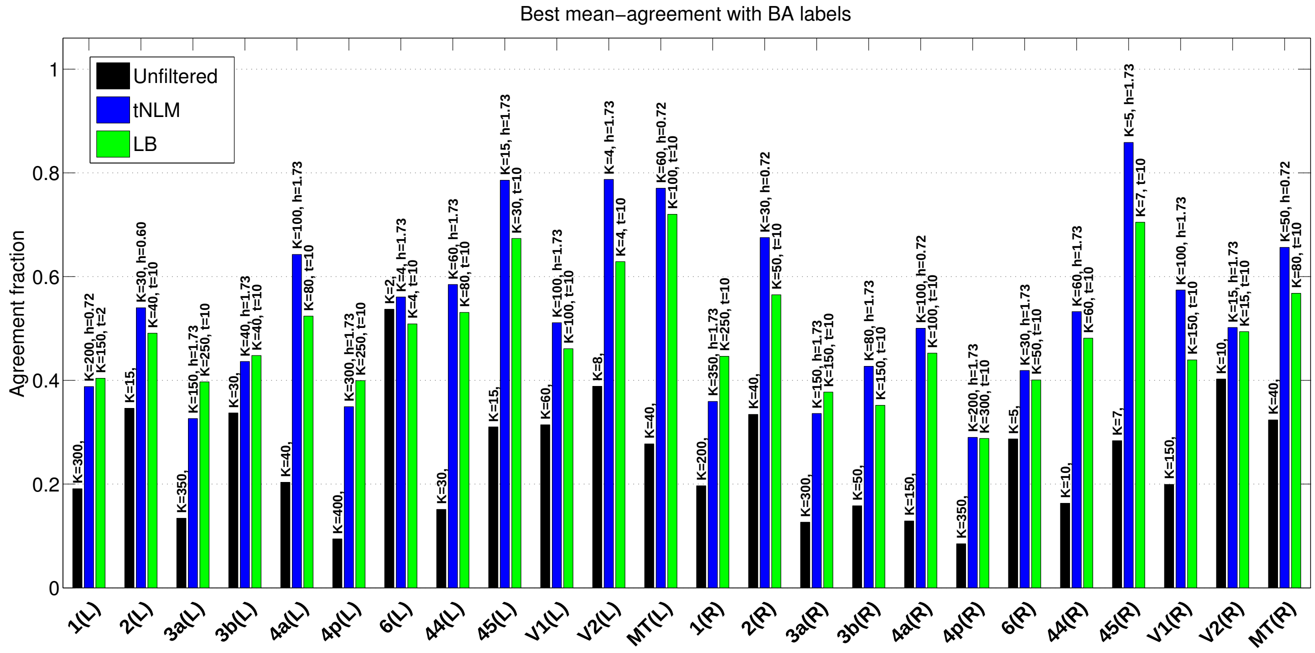

(b)

Figure M: (a) An example of (thresholded) probabilistic Brodmann areas, mapped back to the subject's cortical surface. (b) Best performance of different filtering approaches across different probabilistic Brodmann areas (BA). For each BA and each filtering approach, we select the parameters which achieves the highest mean agreement fraction. The grouped bar plot shows the highest mean agreement fraction and the text on top shows the corresponding parameters. Detailed BA-wise performance results are included in Fig. N.

Brodmann areas	Alternate hypothesis	Signed-rank p-values
1(L)	LB > tNLM	0.0517
2(L)	tNLM > LB	0.00997
3a(L)	LB > tNLM	$4.07{\times}10^{-4}$
3b(L)	LB > tNLM	0.666
4a(L)	tNLM > LB	$2.74{\times}10^{-11}$
4p(L)	LB > tNLM	$1.61{\times}10^{-6}$
6(L)	tNLM > LB	$7.69{\times}10^{-7}$
44(L)	tNLM > LB	0.00243
45(L)	tNLM > LB	$8.77{\times}10^{-7}$
V1(L)	tNLM > LB	0.00861
V2(L)	tNLM > LB	$1.74{\times}10^{-8}$
MT(L)	tNLM > LB	0.000313
PH(L)	tNLM > LB	0.00521
1(R)	LB > tNLM	$2.77{\times}10^{-11}$
2(R)	tNLM > LB	$6.49{\times}10^{-10}$
3a(R)	LB > tNLM	0.00121
3b(R)	tNLM > LB	$9.9{\times}10^{-19}$
4a(R)	tNLM > LB	$2.07{\times}10^{-4}$
4p(R)	tNLM > LB	0.272
6(R)	tNLM > LB	0.07
44(R)	tNLM > LB	$6.91{\times}10^{-4}$
45(R)	tNLM > LB	$5.25{\times}10^{-10}$
V1(R)	tNLM > LB	$2.84{\times}10^{-14}$
V2(R)	LB > tNLM	0.865
MT(R)	tNLM > LB	$3.07{\times}10^{-4}$
PH(R)	tNLM > LB	0.146

Table 1: Statistical tests for higher agreement with probabilistic Brodmann areas: Table of (uncorrected) p-values for one-sided tests for 'best' performance of LB and tNLM filtering (parameters of 'best' parcellation are reported in Fig. M(b)). The procedure and description of the non-parametric tests are same as that of task labels.

We also computed quantitative agreement of N-cuts parcellations with probabilistic BAs. Fig. M(b) shows the summary of the best performances for different filtering approaches for each BA. Consistent with our previous observation for task fMRI, tNLM achieves the highest agreement fraction across several BA (for 18 out of 24 BAs). We also performed the non-parametric statistical tests with the same null hypothesis as the task analysis. The results and alternate hypothesis of the statistical tests are reported in table 1 which reveal that tNLM filtering significantly improves the agreement of several BA labels with the parcellation results as compared with LB filtering. There are few BA labels, such as BA 3a (L), for which LB parcellations shows higher median agreement than tNLM. More complete data are included in Fig. N in which mean agreement is plotted for each method and parameter for each of the BAs.

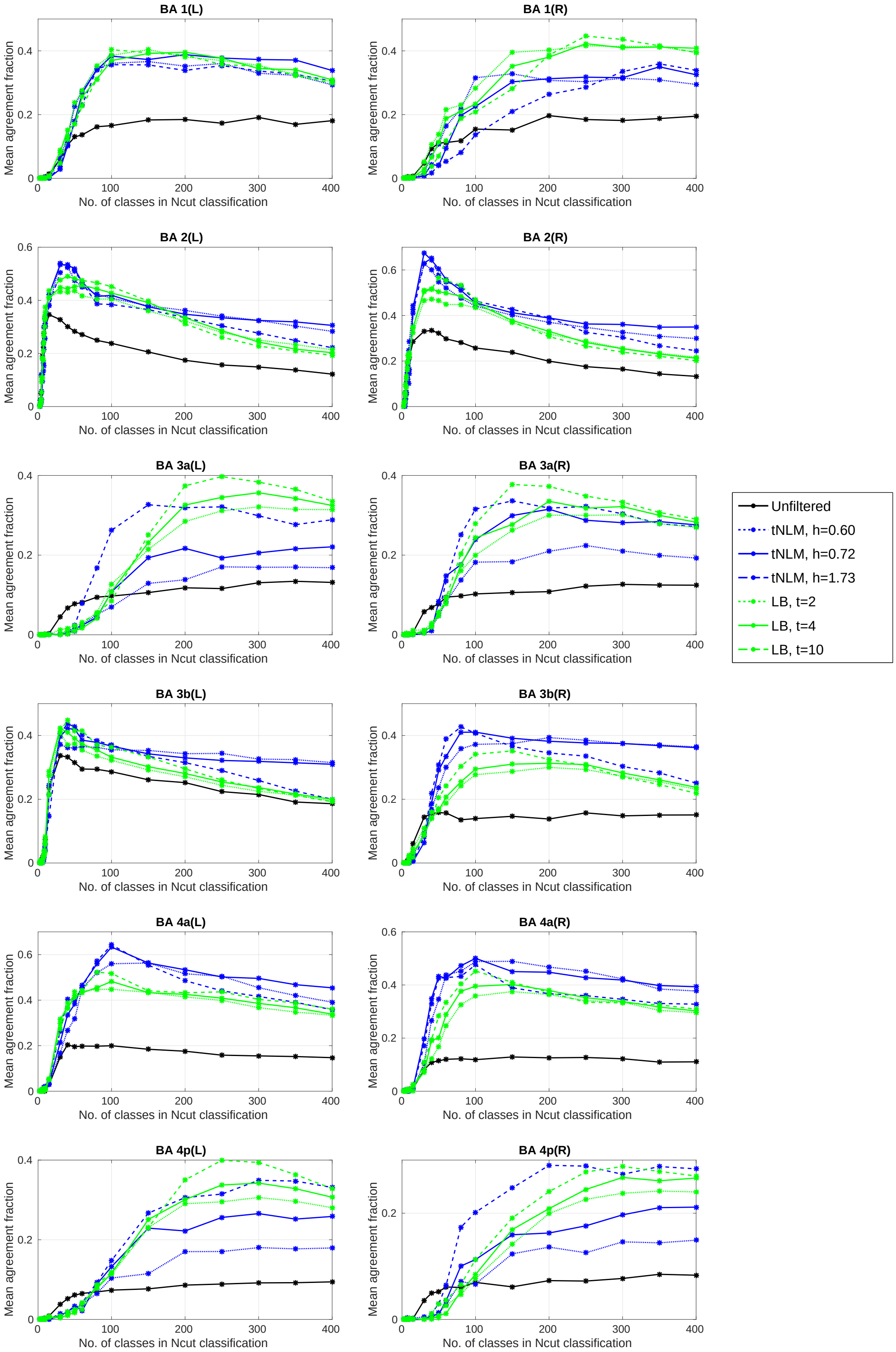

Figure N: Probabilistic Brodmann area based evaluation: Detailed performance of all filtering approaches across several parameters for Brodmann areas (BAs). Line plots show the mean agreement of N-cuts clusters with BAs across 40 subjects with 4 sessions each ($40 \times 4 = 160$ N-cuts clustering results). Left and right column shows agreement for BAs in left and right hemisphere respectively. (Contd. to next page)

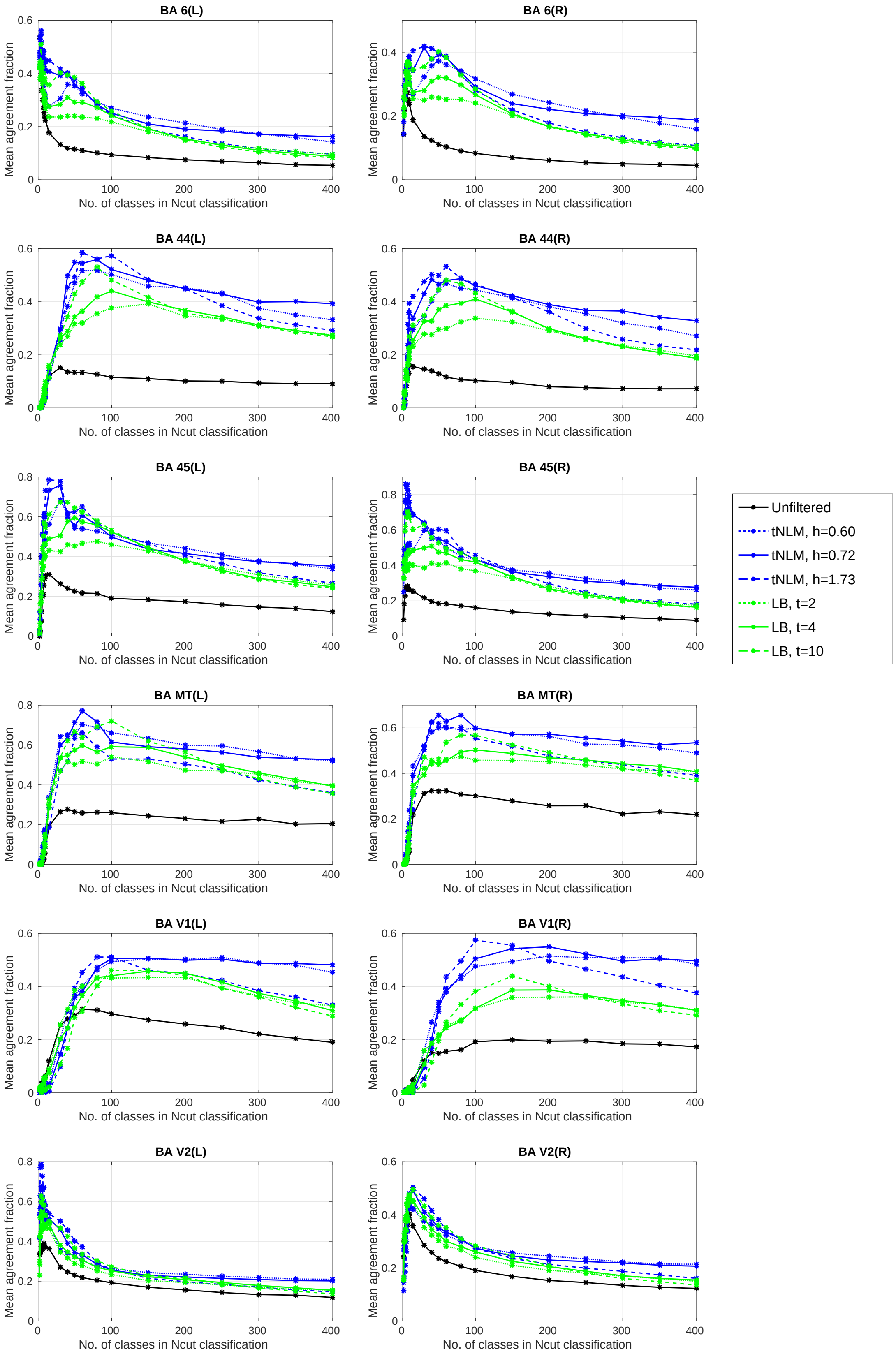

Figure N: (Contd. from previous page)

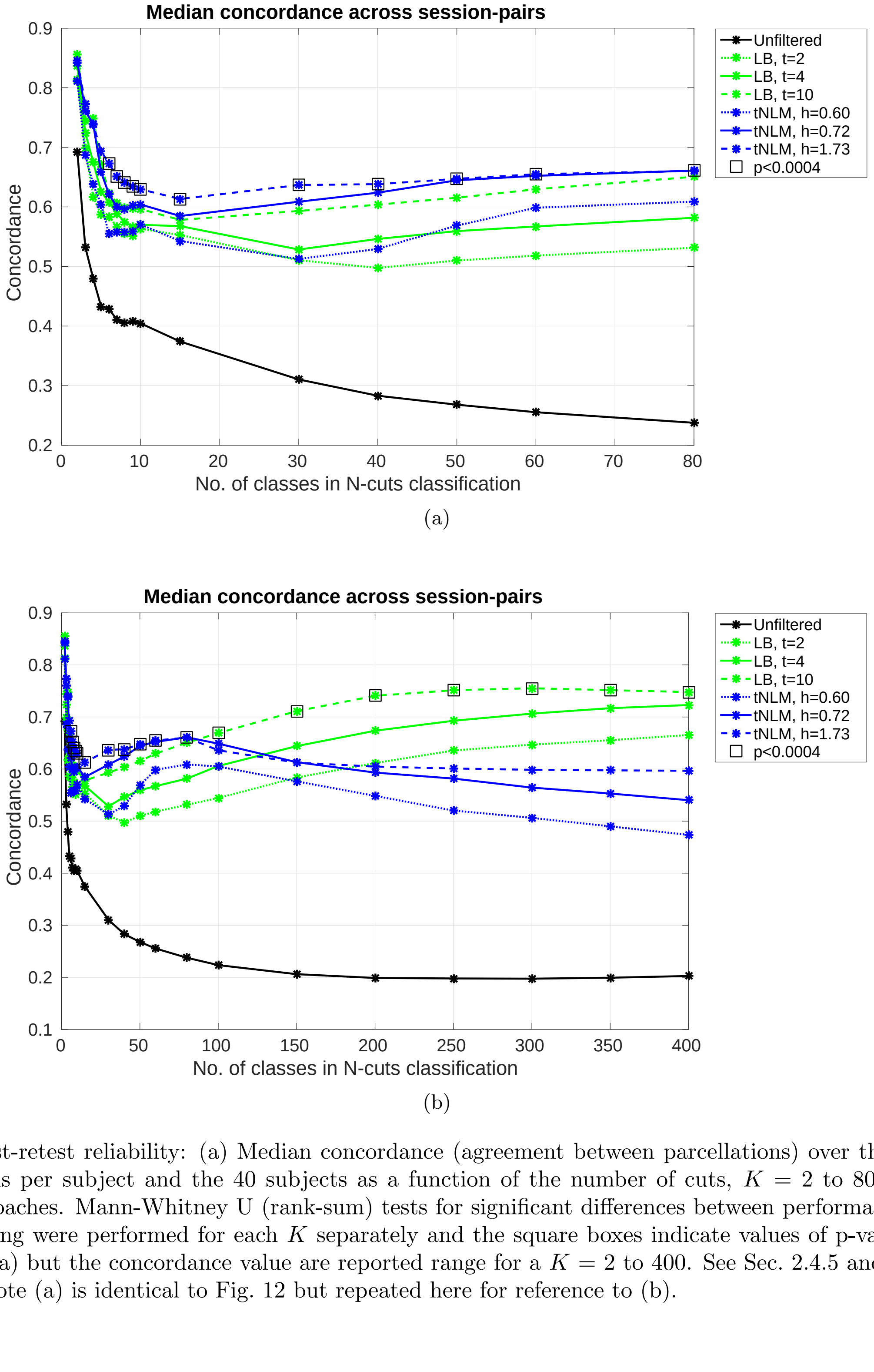

Figure O: Test-retest reliability: (a) Median concordance (agreement between parcellations) over the six pairs of rfMRI sessions per subject and the 40 subjects as a function of the number of cuts, $K = 2$ to 80, for different filtering approaches. Mann-Whitney U (rank-sum) tests for significant differences between performances of tNLM and LB filtering were performed for each K separately and the square boxes indicate values of p-value < 0.0004. (b) Same as (a) but the concordance value are reported range for a $K = 2$ to 400. See Sec. 2.4.5 and 3.6 in paper for details. Note (a) is identical to Fig. 12 but repeated here for reference to (b).

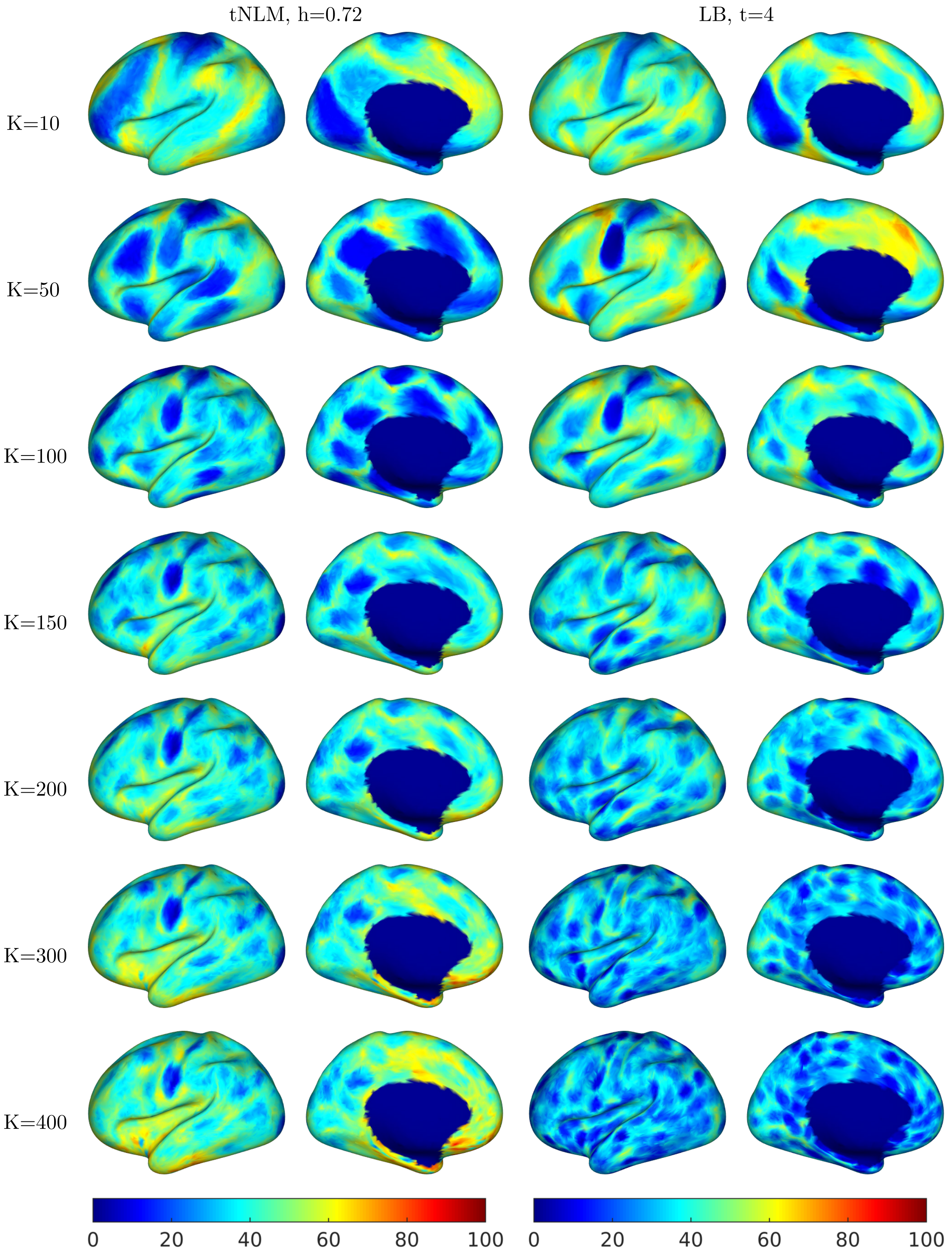

Figure P: Test-retest variability: Cortical maps of frequency of disagreement between parcellations across independent rfMRI sessions of same subject (left) with tNLM filtering (h=0.72) and (right) with LB filtering (t=4). The value at each vertex represents the percentage of times (over 40 subjects $\times$ 6 sessions-pairs) that vertex had been assigned different labels across N-cuts parcellations using rfMRI data from a pair of independent sessions. Also, notice the corresponding size of the N-cuts clusters in Fig. F which would affect the parcellation matching. See Fig. Q for several similar plots with $K \leq 80$.

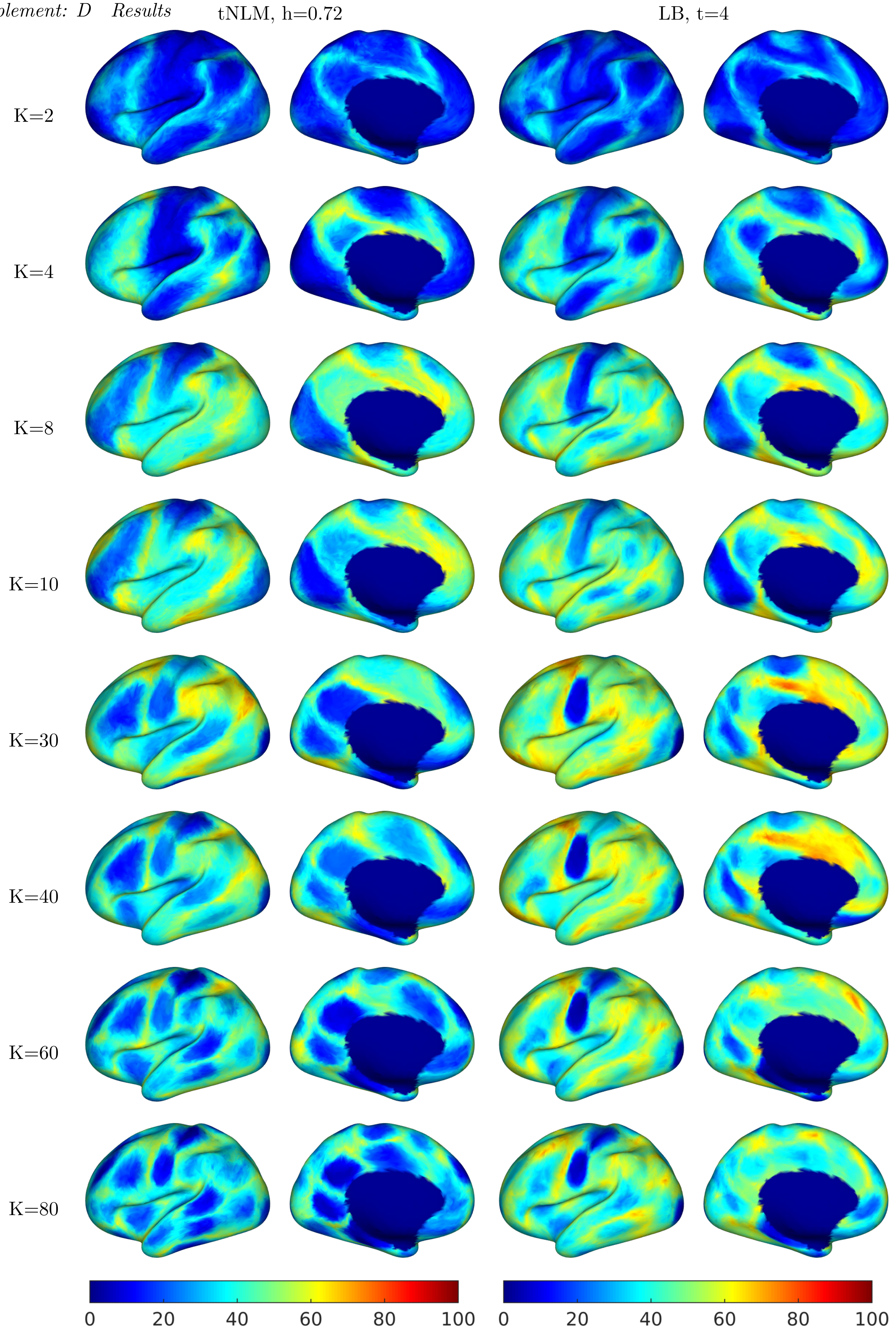

Figure Q: Test-retest variability: (Same as Fig. P but with N-cuts classes $K \leq 80$) Cortical maps of frequency of disagreement between parcellations across independent rfMRI sessions of same subject (left) with tNLM filtering (h=0.72) and (right) with LB filtering (t=4).

References

[1] D. C. Van Essen, S. M. Smith, D. M. Barch, T. E. Behrens, E. Yacoub, and K. Ugurbil, "The WU-Minn Human Connectome Project: An overview," *NeuroImage*, vol. 80, no. 0, pp. 62 – 79, 2013.

[2] WU-Minn Consortium Human Connectome Project, *WU-Minn HCP 500 Subjects + MEG2 Data Release: Reference Manual*, Nov. 2014.

[3] S. M. Smith, C. F. Beckmann, J. Andersson, E. J. Auerbach, J. Bijsterbosch, G. Douaud, E. Duff, D. A. Feinberg, L. Griffanti, M. P. Harms, M. Kelly, T. Laumann, K. L. Miller, S. Moeller, S. Petersen, J. Power, G. Salimi-Khorshidi, A. Z. Snyder, A. T. Vu, M. W. Woolrich, J. Xu, E. Yacoub, K. Uğurbil, D. C. Van Essen, and M. F. Glasser, "Resting-state fMRI in the Human Connectome Project," *NeuroImage*, vol. 80, no. 0, pp. 144 – 168, 2013.

[4] S. Angenent, S. Haker, A. Tannenbaum, and R. Kikinis, "On the Laplace-Beltrami operator and brain surface flattening," *IEEE Transactions on Medical Imaging*, vol. 18, pp. 700–711, Aug 1999.

[5] A. A. Joshi, D. W. Shattuck, P. M. Thompson, and R. M. Leahy, "A parameterization-based numerical method for isotropic and anisotropic diffusion smoothing on non-flat surfaces," *IEEE Transactions on Image Processing*, vol. 18, pp. 1358–1365, June 2009.

[6] S. Seo, M. K. Chung, and H. K. Vorperian, "Heat kernel smoothing using Laplace-Beltrami eigenfunctions," in *Medical Image Computing and Computer-Assisted Intervention (MICCAI)* (T. Jiang, N. Navab, J. Pluim, and M. Viergever, eds.), vol. 6363 of *Lecture Notes in Computer Science*, pp. 505–512, Springer Berlin Heidelberg, 2010.

[7] S. Seo and M. K. Chung, "Laplace-Beltrami eigenfunction expansion of cortical manifolds," in *IEEE International Symposium on Biomedical Imaging: From Nano to Macro*, pp. 372–375, March 2011.

[8] S. Rosenberg, *The Laplacian on a Riemannian manifold: An introduction to analysis on manifolds.* London Mathematical Society Student Texts(No. 31), Cambridge University Press, New York, January 1997.

[9] D. Gale and L. S. Shapley, "College admissions and the stability of marriage," *The American Mathematical Monthly*, vol. 69, pp. 9–15, Jan 1962.

[10] K. Zilles and K. Amunts, "Centenary of Brodmann's map – conception and fate," *Nature Reviews Neuroscience*, vol. 11, pp. 139–145, February 2010.

[11] B. Fischl, N. Rajendran, E. Busa, J. Augustinack, O. Hinds, B. T. Yeo, H. Mohlberg, K. Amunts, and K. Zilles, "Cortical folding patterns and predicting cytoarchitecture," *Cerebral Cortex*, vol. 18, no. 8, pp. 1973–1980, 2008.

[12] D. C. Van Essen, M. F. Glasser, D. L. Dierker, J. Harwell, and T. Coalson, "Parcellations and hemispheric asymmetries of human cerebral cortex analyzed on surface-based atlases," *Cerebral Cortex*, vol. 22, no. 10, pp. 2241–2262, 2012.
